# Burden of diseases and injuries attributable to alcohol consumption in the Middle East and North Africa region, 1990–2019

**DOI:** 10.1038/s41598-022-22901-x

**Published:** 2022-11-11

**Authors:** Saeid Safiri, Seyed Aria Nejadghaderi, Maryam Noori, Mark J. M. Sullman, Gary S. Collins, Jay S. Kaufman, Ali-Asghar Kolahi

**Affiliations:** 1 Road Traffic Injury Research Center, Tabriz University of Medical Sciences, Tabriz, Iran; 2 Department of Community Medicine, Faculty of Medicine, Tabriz University of Medical Sciences, Tabriz, Iran; 3 Research Center for Integrative Medicine in Aging, Aging Research Institute, Tabriz University of Medical Sciences, Tabriz, Iran; 4 Systematic Review and Meta-Analysis Expert Group (SRMEG), Universal Scientific Education and Research Network (USERN), Tehran, Iran; 5 Student Research Committee, School of Medicine, Iran University of Medical Sciences, Tehran, Iran; 6 Urology Research Center, Tehran University of Medical Sciences, Tehran, Iran; 7 Department of Life and Health Sciences, University of Nicosia, Nicosia, Cyprus; 8 Department of Social Sciences, University of Nicosia, Nicosia, Cyprus; 9 Centre for Statistics in Medicine, NDORMS, Botnar Research Centre, University of Oxford, Oxford, UK; 10 NIHR Oxford Biomedical Research Centre, Oxford University Hospitals NHS Foundation Trust, Oxford, UK; 11 Department of Epidemiology, Biostatistics and Occupational Health, Faculty of Medicine, McGill University, Montreal, QC Canada; 12 Social Determinants of Health Research Center, Shahid Beheshti University of Medical Sciences, Tehran, Iran

**Keywords:** Risk factors, Epidemiology, Epidemiology

## Abstract

Alcohol consumption is associated with a number of diseases and injuries, including cardiovascular diseases, cancers, mental and neurological disorders, as well as transport-related injuries. This article reports the alcohol-attributable burden of diseases and injuries at the regional and national levels in the Middle East and North Africa (MENA) region between 1990 and 2019, by sex, age, underlying cause, and Socio-demographic Index (SDI). The regional deaths and disability-adjusted life-years (DALYs) attributable to alcohol consumption were reported for the MENA region, between 1990 and 2019, using the methodological framework and analytical strategies adopted by the Global Burden of Disease (GBD) study 2019. The estimates were all reported as counts, population-attributable fractions, and age-standardised rates per 100,000 population, along with their corresponding 95% uncertainty intervals (UIs). Also, the average annual percentage changes were used to represent the trends of age-standardised rates. In 2019, there were an estimated 22.0 thousand deaths (95% UI: 16.1–29.4) and 1.1 million DALYs (0.8–1.3) attributable to alcohol consumption in the MENA region. The number of DALYs attributable to alcohol consumption were much higher in men (878.0 thousand, 691.4–1104.8) than among women (181.8, 138.6–232.0). The overall age-standardised death and DALY rates attributable to alcohol consumption decreased by 34.5% (13.2–48.3) and 31.9% (16.9–42.5), respectively, over the study period. Egypt (10.1 [5.7–16.6]) and Kuwait (1.1 [0.8–1.5]) had the highest and lowest age-standardised death rates attributable to alcohol consumption, respectively. In 2019, the number of deaths and DALYs in the MENA region were highest in those aged 60–64 and 50–54 years, respectively. A negative association was observed between a country’s SDI and their corresponding age-standardised DALY rates over the period 1990 to 2019. Digestive diseases were the main contributor to the alcohol-attributable burden. Over 1990–2019, the regional deaths and DALYs of diseases and injuries attributable to alcohol consumption decreased with AAPC of − 1.45 (− 1.78 to − 1.12) and − 1.31 (− 1.46 to − 1.15), respectively. The death and DALY rates attributable to alcohol consumption in the MENA region have decreased over the past three decades. Further decreases can be facilitated by implementing country-level policies and increasing public awareness.

## Introduction

Alcohol consumption is associated with more than 200 diseases and injuries^1^, and thus the recommended safe level of alcohol consumption is zero^2^. Evidence shows that the average volume of alcohol consumption has a causal relationship with a number of cancers, cardiovascular diseases, metabolic disorders, respiratory infections, perinatal complications, mental disorders, and neurological consequences^3^. Moreover, in 2016 alcohol consumption disorder was reported to be the most common type of substance use disorder^4^, for which the World Health Organization (WHO) has developed a number of programs^5^.

Alcohol contributes substantially to the overall global burden of diseases, including 4% of all deaths and 4–5% of all disability-adjusted life-years (DALYs). Moreover, alcohol consumption is considered to be one of the most important preventable risk factors. Younger consumers experience more harmful effects than older consumers, partly due to the fact that the injuries caused by alcohol affect younger individuals’ more than older adults. Furthermore, it has been noted that less wealthy individuals and low income countries are at a higher risk, per unit of alcohol consumed, than those from high income countries^6^. Previous research at the global level has shown that in 2016 the alcohol attributable disease burden peaked in the 25–29 age group and was negatively related to the human development index (HDI)^7^. The Middle East and North Africa (MENA) region consists of many low and middle income countries, and in 2016 the age-standardised point prevalence of alcohol consumption was 593.0 (95% uncertainty interval (UI): 507.9–683.0) per 100,000 population. Moreover, alcohol consumption was responsible for 264.6 (204.8 − 342.2) age-standardised DALYs, which comprised 0.8% of the total DALYs in this region^4^.

Previous research reported the burden of diseases attributable to alcohol and drug use over the period 1990–2016, at the global, regional, and national levels^4^, while a second study reported the deaths and DALYs attributable to alcohol consumption between 2000 and 2016^7^. However, both of these studies used data from the Global Burden of Disease (GBD) 2016 study, which is now out-of-date, and neither focused solely on the MENA region to investigate the alcohol-attributable burden of diseases.

In this article, we aimed to address these gaps and to provide the most up-to-date estimates of alcohol consumption for the 21 countries and territories located in the MENA region. The present study used data from the Global Burden of Disease (GBD) 2019 study to report the numbers and age-standardised rate of deaths and DALYs for the diseases and injuries attributable to alcohol consumption by sex, age, underlying cause, and Socio-demographic Index (SDI), from 1990 to 2019.

## Methods

### Overview

The Institute for Health Metrics and Evaluation (IHME) manages the GBD project, which has so far estimated the level and trends associated with 369 diseases and injuries and 87 risk factors, from 1990 to 2019, in 204 countries and territories^8,9^. The present study reports the disease burden attributable to alcohol consumption in the countries that comprise the MENA region, from 1990 to 2019. The MENA region includes: Afghanistan, Algeria, Bahrain, Egypt, Iran, Iraq, Jordan, Kuwait, Lebanon, Libya, Morocco, Oman, Palestine, Qatar, Saudi Arabia, Sudan, the Syrian Arab Republic, Tunisia, Turkey, the United Arab Emirates and Yemen. The total population of MENA was estimated to be 608.7 million in 2019^10^. The Comparative Risk Assessment approach was used in GBD 2019 to estimate the burden of diseases and injuries attributable to alcohol consumption. Detailed information on each analytical step and the methodology used for estimating the burden of diseases, injuries and risk factors can be found in a previous article^8^. The data for the fatal and non-fatal estimates are available at https://vizhub.healthdata.org/gbd-compare/ and http://ghdx.healthdata.org/gbd-results-tool.

### Definition of alcohol consumption

Alcohol consumption (exposure) was defined as the number of grams of pure alcohol consumed per day by current drinkers. The level of exposure was estimated using the following indicators: (a) the proportion of current drinkers, which were those individuals who consumed one or more alcoholic beverage (or an approximation) in the previous year. (b) alcohol consumption, which was the average number of grams of alcohol consumed each day by current drinkers over a one-year period, and (c) litres of alcohol stock per capita, which was the number of litres of pure alcohol per capita over a one-year period. Furthermore, to account for several types of bias, the following variables were also included: (a) number of tourists, which was the total number who visited a location in the previous year. (b) tourists’ duration of stay, which was the total number of days the tourists stayed in the host country, and (c) unrecorded alcohol stock, which was the estimated proportion of the alcohol stock that was made outside the established markets^8^.

### Data sources

A systematic review of the literature was conducted by IHME to identify and extract data on the primary indicators. Population-level survey data, which contained participant-level information, was searched for using the Global Health Exchange (GHDx) and IHME’s online database of health-related data, in order to create the required alcohol consumption indicators on current drinkers and alcohol consumption. The appropriate information was extracted from the data sources^8^, but only data from representative samples were included.

The estimated prevalence of current drinkers was split by age and sex. First, a meta-regression tool (MR-BRT) was used to estimate the region-specific sex ratios to separate the prevalence reported by sex. Spatiotemporal Gaussian Process Regression (ST-GPR) was then used to separate the estimates into the standard GBD five-year age groups, in studies that did not report estimates in these groups. MR-BRT was also used to adjust the data for studies that did not use the standard definition of alcohol consumption^8^.

Since GBD 2017, substantial improvements have been made to the modelling of supply-side-level data. The raw data included the domestic (*WHO|Global Information System on Alcohol and Health (GISAH); Food and Agriculture Organization (FAO)*) and retail supply (*Euromonitor*) of pure ethanol consumed (number of litres). Domestic supply was defined as the summation of the production and imports, minus the exports. The WHO and FAO sources were combined, with the FAO data only being used where there was no WHO data available for that location-year, since the WHO figures already consider the FAO values. The outliers, implausible data points or data that created implausible model fluctuations were removed, with a detailed description of this process being previously reported^8^.

### Modelling strategy

Although population-based studies provide accurate estimates regarding the prevalence of current drinkers, they also underestimate real alcohol consumption levels^8,11^. Therefore, the litres per capita variable was thought to provide a more accurate estimate of the overall volume of alcohol consumption, although this does not produce the age- and sex-specific consumption estimates required to estimate the alcohol attributable burden of diseases and injuries. Therefore, population-based survey data and the total volume of alcohol consumed within a location (sourced from the FAO, GISAH, and Euromonitor) were used to model the patterns of consumption by age and sex^8^.

The survey data was used by ST-GPR to calculate estimates for each location/year/age/sex, as well as to model the alcohol litres per capita (LPC) and the total number of tourists. The LPC were adjusted to take into account unrecorded consumption and consumption by tourists. Furthermore, ST-GPR was also used to generate sex- and age-specific estimates using the estimated percentage of current drinkers within a location/year/sex/age and the consumption trends previously estimated. All estimates were reported as the number of grams/day^8^.

### Data on the estimated relative risk

The theoretical minimum-risk exposure level (TMREL) to alcohol was calculated, which is the level of exposure that minimises the chances of suffering burden from any alcohol-related cause. IHME conducted a systematic literature review to find all cohort and case–control studies that reported a relative risk, hazard ratio, or odds ratio for any alcohol-disease or alcohol-injury pairs that were covered by GBD 2019^8^. The systematic review only included studies that were representative of their specific population, reported a categorical or continuous dose of alcohol consumption, and reported measures of uncertainty. The studies were then used to compute a dose–response model using the Disease Modelling Ordinary Differential Equation (DisMod ODE) solver to fit non-linear Bayesian meta-regressions. A DisMod ODE model was preferred over the conventional mixed effects meta-regression, as it is able to estimate nonparametric splines over doses (i.e., there is a non-linear dose–response relationship) and to include heterogeneous doses through dose integration (i.e., research normally reports doses categorically in wide ranges and DisMod ODE estimates specific doses when the categories overlap between studies, using an integration step). The results of the meta-regression were then used to estimate a non-parametric curve for all doses between zero and 150 g/day, as well as their corresponding relative risks. The relative risk was considered to be the same for all-ages and for both males and females^8^.

The systematic review^8^ found the following diseases and injuries to be associated with alcohol consumption: cardiovascular diseases, diabetes and kidney diseases, digestive diseases, neoplasms, neurological disorders, respiratory infections and tuberculosis, self-harm and interpersonal violence, substance use disorders, transport injuries and unintentional injuries.

### Estimation of the proportion of cancers attributable to alcohol consumption

The population-attributable fraction (PAF) was used to estimate the burden of diseases and injuries related to alcohol consumption by country, age, sex, and year, using the following formula:

$$PAF(x) = \frac{{P_{A} + \int_{0}^{150} {P(x)*RR_{C} (x)dx - 1} }}{{P_{A} + \int_{0}^{150} {P(x)*RR_{C} (x)dx} }}\;\;\;\;\;\;P(x) = P_{C} *\Gamma (p)$$

where: P_c_ denotes the prevalence of current drinkers, P_a_ is the prevalence of abstainers, RR_C_(x) is the relative risk function for current drinkers, and *p* is the parameters determined by the mean and standard deviation of exposure. This equation was used to produce 1000 draws of the exposure and relative risk models and the PAF was used to calculate the attributable burden^8^.

The deaths and DALYs attributable to alcohol consumption for each country, age, sex, year, and disease/injury were calculated by multiplying the PAFs with the estimated number of deaths or DALYs for each country, age, sex, year, and disease/injury. The number of deaths were estimated using the Cause of Death Ensemble model (CODEm) in GBD 2019. CODEm generates a number of individual models to identify the best model fit using all obtainable data and covariates. The predictive validity for the different models, or combinations of models, were assessed and the one with the best (out-of-sample) predictive validity was chosen. Furthermore, the DALYs for diseases/injuries were estimated in three stages: (a) the years lived with disability (YLDs) was calculated by multiplying the severity-specific disability weights with the prevalence of each severity category for each disease/injury; (b) the number of deaths in an age group were then multiplied by the remaining life expectancy in that age group, taken from the GBD standard life table, to produce the years of life lost (YLLs) for each disease/injury; (c) the YLLs and YLDs were then summed for each disease/injury to calculate the DALYs. A comprehensive description of the methods used to calculate the total number of deaths and DALYs has been previously reported^8^. The estimates were all reported as counts, proportions (PAFs) and age-standardised rates per 100,000, along with their corresponding 95% UIs. The UIs were calculated by running 1000 repetitions at each computational step and through the inclusion of uncertainty from multiple sources (i.e., input data, measurement error and estimates of residual non-sampling error). The UIs were defined as the 25th and 975th values of the ordered draws^8^.

The relationship between the burden of diseases/injuries attributable to alcohol consumption and SDI was also investigated. Smoothing splines models^12^ were used to determine the shape of the association between the burden attributable to alcohol consumption, in terms of DALYs, and SDI for the 21 countries in MENA. SDI is a multifactorial measure of socio-economic development which contains the lag-distributed income per capita, educational attainment for the population aged > 15, and the total fertility rate < 25 years old. The SDI ranges from the least developed (0) to the most developed (1). The age-standardised death and DALY rates were mapped using R software (version 3.5.2). In addition, annual percentage change (APC) and the average APC (AAPC) were calculated using the joinpoint regression tool^13,14^.

### Ethics approval

The present study was approved by ethics committee of Shahid Beheshti University of Medical Sciences (IR.SBMU.RETECH.REC.1401.070). This study is based on publicly available data and solely reflects the opinion of its authors and not that of the Institute for Health Metrics and Evaluation.

## Results

### The Middle East and North Africa region

In 2019, there were an estimated 22.0 thousand deaths (95% UI: 16.1 to 29.4) attributable to alcohol consumption in the MENA region, representing 0.7% (0.5 to 0.9) of all deaths (Table 1). Also in 2019, there were 18.7 (13.7 to 25.4) thousand deaths in men and 3.2 (2.1 to 4.8) thousand deaths in women (Table S1). The age-standardised death rate associated with alcohol consumption in 2019 (4.6 [3.4 to 6.3] per 100,000) was 34.5% lower (13.2 to 48.3) than the 1990 level (7.1 [5.3 to 9.1]) (Table S2). During 2019, alcohol consumption caused 1.1 million DALYs (0.8 to 1.3), representing 0.6% (0.5 to 0.8) of all DALYs in both sexes combined, with 878.0 (691.4 to 1104.8) thousand DALYs in men and 181.8 (138.6 to 232.0) thousand DALYs in women (Table S3). Between 1990 and 2019, the age-standardised DALY rate (per 100,000) decreased from 276.6 (218.9 to 339.0) to 188.3 (148.5 to 235.7), a relative decrease of 31.9% (16.9 to 42.5) from the 1990 level (Table S4).

**Table 1:** The burden of diseases attributable to alcohol consumption in the Middle East and North Africa region in 2019 and percentage change of age-standardised rates during 1990–2019 (Generated from data available from http://ghdx.healthdata.org/gbd-results-tool).

	Deaths (95% UI)				DALY (95% UI)			
	Counts (2019)	PAF (2019)	ASRs (2019)	% change in ASRs 1990–2019	Counts (2019)	PAF (2019)	ASRs (2019)	% change in ASRs 1990–2019
North Africa and Middle East	21,980 (16,085, 29,408)	0.7 (0.5, 0.9)	4.6 (3.4, 6.3)	− 34.5 (− 48.3, − 13.2)	1,059,791 (842,123, 1,316,847)	0.6 (0.5, 0.8)	188.3 (148.5, 235.7)	− 31.9 (− 42.5, − 16.9)
Afghanistan	583 (375, 853)	0.2 (0.2, 0.3)	3.8 (2.5, 5.4)	− 23.1 (− 43.9, 4.2)	35,540 (25,680, 47,682)	0.2 (0.2, 0.3)	155.6 (110.4, 208.9)	− 14.3 (− 33.5, 12.8)
Algeria	1285 (766, 1972)	0.6 (0.4, 1)	3.3 (1.9, 5.3)	5.8 (− 36.8, 82.3)	72,122 (51,488, 96,915)	0.7 (0.5, 0.9)	170.2 (119.7, 231.3)	1.8 (− 28.5, 42.9)
Bahrain	69 (44, 101)	1.6 (1.1, 2.3)	5.1 (3, 8)	− 76.2 (− 85.7, − 62.3)	3859 (2757, 5297)	1.4 (1, 1.8)	227.4 (159.7, 320.2)	− 67.5 (− 77.6, − 54.7)
Egypt	6207 (3373, 10,443)	1.1 (0.6, 1.8)	10.1 (5.7, 16.6)	− 5.6 (− 46.7, 57.4)	221,195 (135,795, 354,970)	0.8 (0.5, 1.3)	290.3 (177, 457.1)	− 6.3 (− 45.9, 58)
Iran (Islamic Republic of)	2976 (2244, 3996)	0.8 (0.6, 1)	3.8 (2.9, 5.2)	36.7 (2.7, 89.6)	139,833 (109,766, 175,225)	0.7 (0.6, 0.9)	158.9 (124.4, 200.3)	27 (3.6, 59.5)
Iraq	817 (406, 1418)	0.5 (0.2, 0.8)	2.5 (1.1, 4.6)	− 70.6 (− 87.2, − 42.9)	54,549 (35,290, 82,758)	0.5 (0.4, 0.8)	143 (88, 225.5)	− 61.5 (− 76.7, − 37.9)
Jordan	186 (97, 309)	0.6 (0.3, 0.9)	2.3 (1.2, 4)	− 16.5 (− 56.6, 65.5)	12,615 (8213, 17,650)	0.6 (0.4, 0.8)	121.4 (77.1, 176.4)	− 12.6 (− 43, 36.8)
Kuwait	32 (23, 45)	0.3 (0.2, 0.4)	1.1 (0.8, 1.5)	− 14.5 (− 34.1, 8.9)	3363 (2394, 4598)	0.4 (0.3, 0.6)	68.1 (49.9, 90)	− 10.8 (− 24.5, 4)
Lebanon	262 (147, 419)	0.8 (0.5, 1.2)	5 (2.8, 8)	− 64.3 (− 78.3, − 46.1)	10,835 (7429, 15,522)	0.8 (0.6, 1.1)	203.8 (138.8, 291.2)	− 61.9 (− 73.9, − 47.1)
Libya	209 (124, 310)	0.7 (0.4, 0.9)	3.6 (2.2, 5.4)	35.9 (− 15.4, 120.4)	11,185 (7345, 15,020)	0.7 (0.5, 0.8)	158.8 (103.5, 214.5)	32.1 (− 8.2, 87.4)
Morocco	1108 (638, 1698)	0.5 (0.3, 0.7)	3.2 (1.8, 5)	− 58.4 (− 73.8, − 36.7)	58,610 (40,044, 82,346)	0.6 (0.4, 0.8)	157.6 (107.7, 222.5)	− 51.5 (− 65.2, − 32.1)
Oman	67 (33, 108)	0.5 (0.3, 0.9)	2.3 (1, 3.9)	− 12.6 (− 47, 62.1)	6057 (4026, 8578)	0.7 (0.5, 1)	125.8 (79.5, 182.1)	− 10.1 (− 34.1, 29.2)
Palestine	219 (155, 298)	1.3 (1, 1.7)	8.6 (5.8, 12.2)	1.9 (− 37.9, 85.6)	9873 (7511, 12,534)	1 (0.8, 1.2)	288.9 (215.2, 375.2)	2.6 (− 30.4, 61.3)
Qatar	75 (49, 110)	1.7 (1.2, 2.3)	6.8 (4.2, 10)	− 26.4 (− 56, 19.7)	5715 (4097, 7660)	1.3 (1, 1.6)	218.1 (152.5, 296.2)	− 37.5 (− 58.5, − 8.7)
Saudi Arabia	722 (352, 1405)	0.6 (0.3, 1)	3.4 (1.8, 6.1)	− 61.9 (− 77.7, − 26.4)	50,542 (30,270, 81,200)	0.6 (0.4, 0.9)	141.3 (86.8, 233.6)	− 54 (− 70.6, − 20.9)
Sudan	494 (300, 774)	0.2 (0.2, 0.4)	2.6 (1.5, 4)	− 74.4 (− 86.5, − 44.6)	29,763 (21,608, 39,519)	0.2 (0.2, 0.3)	105 (75.4, 144.5)	− 71.8 (− 83.3, − 48.3)
Syrian Arab Republic	567 (306, 938)	0.7 (0.4, 1)	4.6 (2.5, 7.7)	− 49.5 (− 70.1, − 22)	24,012 (15,482, 35,496)	0.6 (0.4, 0.8)	172.9 (112.1, 256.6)	− 45.4 (− 62.6, − 24)
Tunisia	723 (446, 1109)	1.1 (0.7, 1.5)	5.6 (3.4, 8.6)	37.1 (− 15.9, 120.2)	34,614 (24,314, 47,583)	1.2 (0.9, 1.5)	271.1 (189.9, 371.3)	23.4 (− 10.1, 70.9)
Turkey	3941 (2551, 5734)	0.9 (0.6, 1.2)	4.3 (2.7, 6.4)	− 35.6 (− 58.8, 1.7)	194,539 (143,449, 256,399)	1 (0.8, 1.2)	208.7 (153.1, 276.7)	− 33.8 (− 50.8, − 11.1)
United Arab Emirates	553 (306, 884)	1.9 (1.1, 2.8)	8.4 (4, 13.8)	− 63.1 (− 81.4, − 37.5)	34,097 (22,366, 49,773)	1.6 (1.1, 2.2)	342 (204.3, 523.2)	− 55.9 (− 73.3, − 31.8)
Yemen	861 (492, 1325)	0.5 (0.3, 0.7)	5.2 (2.9, 8.1)	− 67.9 (− 80.1, − 50.2)	45,794 (30,451, 64,918)	0.4 (0.3, 0.5)	211.5 (137.3, 303.9)	− 61.9 (− 74.6, − 44.1)

*DALY* Disability adjusted life year, *GBD* Global Burden of Disease, *ASRs* Age-standardised rates.

### National level

**Figure 1:**
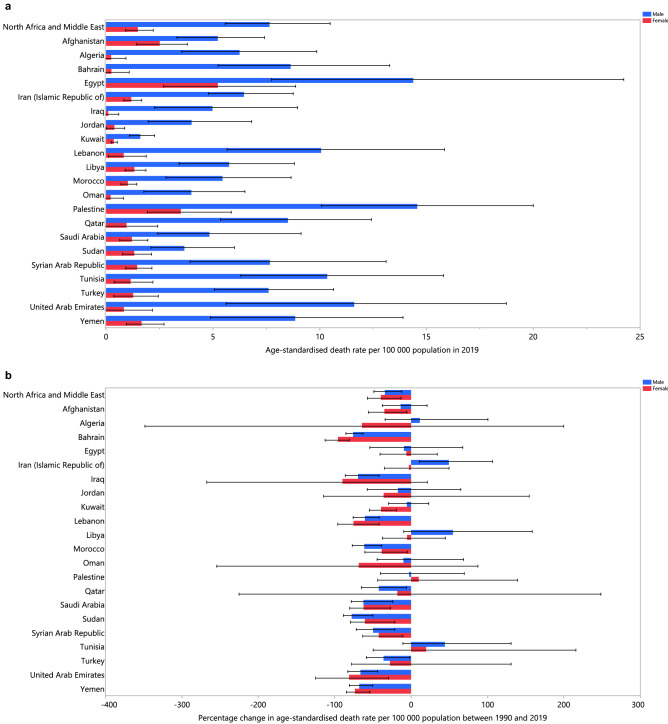
Age-standardised death rate (per 100,000 population) in 2019 (**a**) and percentage change in the age-standardised death rate from 1990 to 2019 (**b**) of disease and injuries attributable to alcohol consumption in the Middle East and North Africa region, by sex and country. (Generated from data available from http://ghdx.healthdata.org/gbd-results-tool).

In 2019, the proportion of all deaths attributable to alcohol consumption varied markedly by country (from 0.2% to 1.9%). The United Arab Emirates (1.9% [1.1 to 2.8]), Qatar (1.7% [1.2 to 2.3]) and Bahrain (1.6% [1.1 to 2.3]) had the three highest PAFs. In contrast, the lowest PAFs were found in Afghanistan (0.2% [0.2 to 0.3]), Sudan (0.2% [0.2 to 0.4]) and Kuwait (0.3% [0.2 to 0.4]) (Table 1). The age-standardised death rate attributable to alcohol consumption in 2019 ranged from 1.1 to 10.1 per 100,000. In 2019, Egypt (10.1 [5.7 to 16.6]), Palestine (8.6 [5.8 to 12.2]) and the United Arab Emirates (8.4 [4.0 to 13.8]) had the three highest age-standardised death rates attributable to alcohol consumption (per 100,000). In contrast, the lowest rates were found in Kuwait (1.1 [0.8 to 1.5]), Oman (2.3 [1.0 to 3.9]) and Jordan (2.3 [1.2 to 4.0]) (Table 1). The age-standardised death rates attributable to alcohol consumption in 2019, for males and females, are presented in Fig. 1a. An increase in the age-standardised death rate attributable to alcohol consumption, from 1990 to 2019, was only observed in Iran (36.7% [2.7 to 89.6]). In contrast, the largest decreases over this period were observed in Bahrain (− 76.2% [− 85.7 to − 62.3]), Sudan (− 74.4% [− 86.5 to − 44.6]) and Iraq (− 70.6% [− 87.2 to − 42.9]) (Table S2). The percentage change in the age-standardised death rates, from 1990 to 2019, for males and females, are presented in Fig. 1b.

The proportion of all DALYs attributable to alcohol consumption in 2019 differed considerably by country (from 0.2% to 1.6%). The United Arab Emirates (1.6% [1.1 to 2.2]), Bahrain (1.4% [1.0 to 1.8]) and Qatar (1.3% [1.0 to 1.6]) had the three highest PAFs. In contrast, the lowest PAFs were found in Afghanistan (0.2% [0.2 to 0.3]), Sudan (0.2% [0.2 to 0.3]) and Yemen (0.4% [0.3 to 0.5]) (Table 1). The age-standardised DALY rate attributable to alcohol consumption in 2019 ranged from 68.1 to 342.0 per 100,000. The United Arab Emirates (342.0 [204.3 to 523.2]), Egypt (290.3 [177.0 to 457.1]) and Palestine (288.9 [215.2 to 375.2]) had the three highest age-standardised DALY rates. In contrast, the lowest rates were found in Kuwait (68.1 [49.9 to 90.0]), Sudan (105.0 [75.4 to 144.5]) and Jordan (121.4 [77.1 to 176.4]) (Table 1). The 2019 age-standardised DALY rates are presented by sex in Fig. S1. Palestine and Egypt had the highest age-standardised DALY rates attributable to alcohol consumption for males and females, respectively, while Kuwait had the lowest DALY rate for both sexes.

An increase in the DALY rate attributable to alcohol consumption, from 1990 to 2019, was only observed in Iran (27.0% [3.6 to 59.5]). In contrast, the largest decreases were observed in Sudan (− 71.8% [− 83.3 to − 48.3]) Bahrain (− 67.5% [− 77.6 to − 54.7]), and Lebanon (− 61.9% [− 73.9 to − 47.1]) (Table 1). The percentage changes in the age-standardised DALY rates, from 1990 to 2019, for males and females are presented in Fig. S2.

### Age and sex patterns

**Figure 2:**
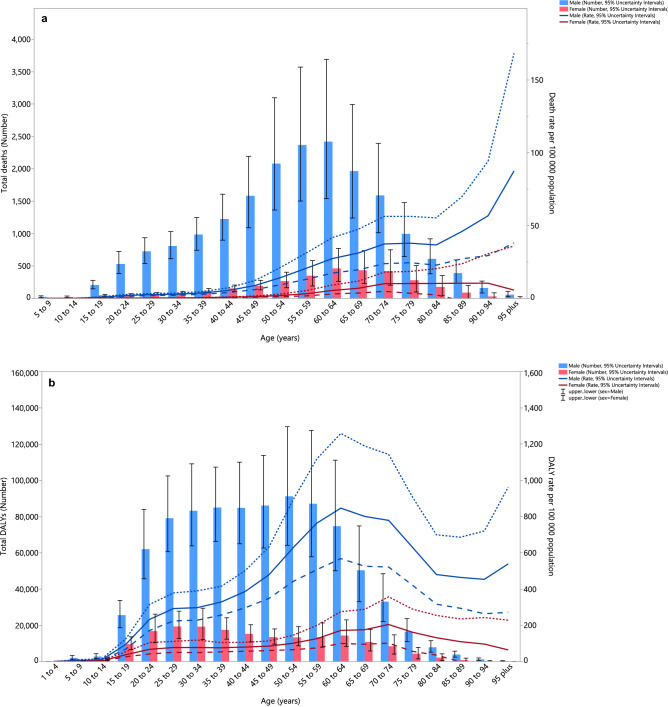
Numbers of deaths and death rate (**a**) and number of DALYs and DALY rate (**b**) of disease and injuries attributable to alcohol consumption in the Middle East and North Africa region, by age and sex in 2019. Dotted and dashed lines indicate 95% upper and lower uncertainty intervals, respectively (Generated from data available from http://ghdx.healthdata.org/gbd-results-tool).

In 2019, the number of deaths attributable to alcohol consumption in the MENA region were highest in the 60–64 age group. The death rate attributable to alcohol consumption started to increase from the 20–24 age group and peaked in the 95^+^ age group for males, and in the 90–94 age group for females. There were substantial differences between males and females, in terms of the number of deaths and the death rate (Fig. 2a). Furthermore, in 2019 the number of DALYs attributable to alcohol consumption in the MENA region were highest in the 50–54 age group for males and in the 25–29 age group for females. For males, the DALY rate started increasing in the early age groups, peaking in the 60–64 age group and then decreased with age. For females, the DALY rate started to increase during the middle ages, with the highest DALY rate being in the 70–74 age group (Fig. 2b).

Among people aged less than 20 years old, the largest increases in the age-standardised death and DALY rates were observed in Libya. In contrast, the largest decrease in the death rate was found in Sudan, while the largest decrease in the DALY rate was found in Lebanon. Regarding the 20–54 age group, Libya had the largest increases in the age-standardised death and DALY rates, whereas Sudan had the largest decreases in both rates. In addition, the largest increases in the age-standardised death and DALY rates were found among individuals aged over 55 years old in Tunisia, while the largest decreases in these rates were found among the same age group in Bahrain (Figs. S3–S4).

### Underlying causes

**Figure 3:**
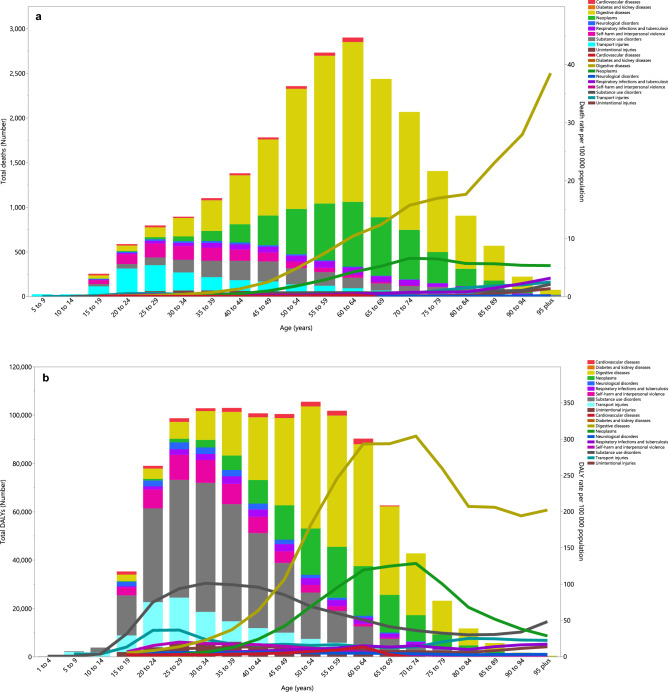
Numbers of deaths and death rate (**a**) and number of DALYs and DALY rate (**b**) of disease and injuries attributable to alcohol consumption in the Middle East and North Africa region, by age and cause in 2019 (Generated from data available from http://ghdx.healthdata.org/gbd-results-tool).

In 2019, the death rate from alcohol-related digestive diseases was higher in people aged 30 and older than for all other causes. The death rate starting increasing in the 35–39 age group and peaked in the 95^+^ age group (Fig. 3a). In addition, the death rate attributable to neoplasms accounted for the second largest proportion, which started increasing in the 45–49 age group and peaked in the 70–74 age group, before decreasing in the remaining age groups (Fig. 3a). The DALY rate attributable to digestive diseases started to increase in the 20–24 age group and peaked in the 70–74 age group, then decreased in the last five age groups. In addition, the DALY rate attributable to neoplasms started to increase in the 30–34 age group and peaked in the 70–74 age group, before decreasing in the remaining age groups. Substance use disorders accounted for the third largest proportion and its DALY rate started to increase in the 10–14 age group and reached its highest level in the 30–34 age group (Fig. 3b).

### Burden of diseases and injuries attributable to alcohol consumption by Socio-demographic Index (SDI)

**Figure 4:**
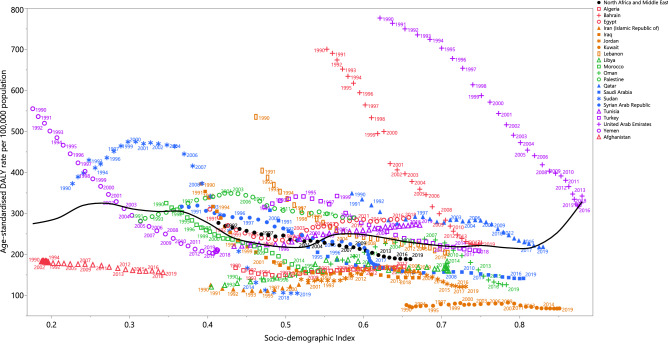
Age-standardised DALY rates of diseases and injuries attributable to alcohol consumption for 21 countries and territories from 1990 to 2019, by SDI; Each point in a line represents the observed age-standardised DALY rate for each year starting at 1990 and ending at 2019. In all countries, SDI has increased over time, so progress in SDI is associated with points further to the right and later years for a given country. Expected values based on the Socio-demographic Index and disease rates in all locations are shown as the black line. *DALY* disability-adjusted-life-years. *SDI* Socio-demographic Index (Generated from data available from http://ghdx.healthdata.org/gbd-results-tool).

There was a generally negative association between a country’s SDI and their corresponding age-standardised DALY rates attributable to alcohol consumption, from 1990 to 2019. Countries higher than the solid black line had a higher than expected burden, while those below the line had a lower than expected burden. Most of the countries showed a decrease in the age-standardised DALY rates from 1990 to 2019. The United Arab Emirates and Bahrain had higher than expected burdens for all years during the measurement period, while lower than expected burdens were found in Kuwait, Iran, Jordan, Libya, Algeria and Afghanistan throughout the measurement period. Qatar, Turkey, Lebanon, Saudi Arabia, Syrian Arabic Republic, Morocco and Yemen had a higher than expected burden at the start of the measurement period, but their burden became lower than expected during the last few years (Fig. 4).

### Joinpoint trends of diseases and injuries attributable to alcohol consumption

Over 1990–2019, the regional deaths and DALYs of diseases and injuries attributable to alcohol consumption decreased with AAPC of − 1.45% (− 1.78 to − 1.12) and − 1.31% (− 1.46 to − 1.15), respectively (Tables S2 and S4). The AAPC of alcohol-attributable deaths in males and females in 2019 were − 1.43% (− 1.76 to − 1.10) and − 1.72% (− 1.82 to − 1.61), respectively, over the measurement period in MENA (Table S1). Also, the AAPC of regional alcohol-attributable DALYs in males and females over 1990–2019 were − 1.35% (− 1.51 to − 1.19) and − 1.28% (− 1.37 to − 1.19) (Table S3). The AAPC of deaths attributable to alcohol consumption decreased in most countries in MENA and ranged from − 4.96% (− 5.59 to − 4.33) in Bahrain to 1.25% (0.52 to 1.98) in Libya (Table S2). Moreover, the AAPC of DALYs attributable to alcohol consumption in MENA ranged from − 4.35% (− 4.81 to − 3.88) in Sudan to 0.97% (0.41 to 1.54) in Libya over 1990–2019 (Table S4).

## Discussion

The present study used pre-existing data from the GBD study 2019 to describe the disease and injury burden due to alcohol consumption in the MENA region. The study found that in 2019 alcohol consumption was responsible for 22.0 thousand deaths and 1.1 million DALYs in this region. Furthermore, we found that there was a 34.5% reduction in the age-standardised death rate with AAPC of − 1.45% and a 31.9% reduction in the DALY rate with AAPC of − 1.31% attributable to alcohol consumption over the period 1990 to 2019. This is in accordance with a 23.3% global reduction in the age-standardised DALY rate due to alcohol consumption^8^. Considering alcohol consumption is a unique risk factor for several injuries and diseases^15,16^, measuring the health effects of alcohol consumption and the successful use of control measures could be potentially helpful in preventing or reducing subsequent health problems^17^.

This study also found that in 2019 0.7% of all deaths and 0.6% of all DALYs were due to alcohol consumption. According to the WHO regions, the proportion of deaths and DALYs caused by alcohol drinking were lowest in the Eastern Mediterranean region^6^, where 0.7% of all deaths and 0.7% of all DALYs were attributable to alcohol consumption^5^. Similarly, using the GBD regional classification, MENA had the lowest alcohol-related age-standardised rates for deaths and DALYs in the world^7^, which is probably due to the fact that MENA also has the lowest alcohol consumption per capita^18^. Furthermore, many countries located in MENA are Muslim majority countries, where the Islamic laws prohibit alcohol production, consumption, transport and trade services. As a result, the importance of religious beliefs and attitudes, along with religion-based alcohol policies in most of the MENA countries, may explain the low alcohol consumption rate^19,20^. At the global level, a recent publication using GBD 2016 data reported that alcohol was responsible for 9.0% and 7.6% of all age-standardised deaths and DALYs, respectively^16^. Furthermore, 5.3% and 5.0% of the global age-standardised deaths and DALYs were reported to be as a result of alcohol consumption, respectively, using data obtained from the WHO Global Health Estimates 2016^7^. The disparities in the reported global PAFs are likely due to variations in the methodologies used in each study.

In 2019, the DALY rate due to alcohol consumption started to rise during adolescence and peaked among older adults. Moreover, the GBD 2019 global risk factor study reported that alcohol consumption was the leading cause of DALYs in the 25–49 age group from 1990 to 2019^8^. Furthermore, alcohol related mortality has been found to occur mainly in young individuals, with more than half (52.4%) of all alcohol-related deaths being found among those younger than 60 years old^7^. This finding highlights the need for more effective preventive measures in this age group. There were also marked differences between men and women in the MENA region, in terms of the number of deaths and DALYs due to alcohol consumption. In line with this, an analysis of 16 general population surveys from 10 countries noted substantial differences between the drinking behaviors of men and women. The level of alcohol consumption was consistently higher among men (than women) in both frequency and quantity, as well as in rates of heavy drinking episodes. In contrast, women were more likely to be life-time abstainers^21^. However, it is projected that the ratio of male to female drinkers will reduce in the Eastern Mediterranean region by 0.006 annually, due in part to increased alcohol consumption among females^18^. In our study, the DALYs attributable to alcohol consumption reduced with AAPC of 1.28% over the last three decades in MENA.

Our study also found that Iran was the only country in the MENA region that showed increases in the age-standardised death and DALY rates attributable to alcohol consumption between 1990 and 2019. Also, Libya and Iran had the highest increase in the AAPC of deaths and DALYs attributable to alcohol consumption in MENA over 1990–2019. A recent systematic review and meta-analysis reported that only one in eight Iranians had ever consumed alcoholic beverages^22^. However, it has also been noted that substandard and adulterated alcoholic drinks are frequently consumed in Iran, due to policies forbidding legal alcohol marketing and sales^23,24^. In addition, as a result of the depreciation in the Iranian currency over recent years, people have been driven to buy handmade alcoholic beverages underground, rather than legally imported high quality products, which may have led to increased levels of alcohol poisoning, death and disability across the country^19^. Although the Iranian Ministry of Health has developed a number of programs aimed at reducing alcohol consumption by 10%, they have not been successfully implemented, due to the low public health service capacity in Iran^19^.

Generally, a negative relationship was noted between the burden due to alcohol consumption and a country’s SDI level. Although not completely comparable, a similar relationship was reported globally with HDI levels, with low HDI countries having the highest age-standardised DALY rates attributable to alcohol consumption^7^. Moreover, a negative relationship was found between the burden caused by alcohol consumption and a country’s income level^5^. Since 1990, alcohol consumption has decreased in most high income countries, particularly in Eastern Europe. During the same time period, alcohol consumption increased markedly in many upper-middle and lower-middle income countries, particularly in China and India^5,18^. This trend is expected to continue across the globe up to 2030^18^. In Sudan, the alcohol-related age-standardised DALY rates peaked over the period 2000–2005, which may be due to psychological distress from the civil war in South Sudan, which started in 1955 and finished in 2005^25,26^.

In 2019, digestive diseases and neoplasms were the two largest causes of death and DALY rates attributable to alcohol consumption in the MENA region. This finding contrasts with the global GBD 2016 findings, where tuberculosis and road injuries were the two most common alcohol-related causes of death for those aged 15–49 years old, while cancers had the largest burden for individuals 50 or older^16^. In line with the fact that MENA has the lowest alcohol consumption per capita^18^, it has also been reported that this region had the lowest death and prevalence rates of cirrhosis and alcohol-related liver disease in the world^27,28^. Conversely, in MENA the largest proportion of deaths and prevalent cases of cirrhosis were caused by hepatitis B and C^27^. It is also important to note that alcohol has a synergistic effect with hepatitis B and hepatitis C infections and this combination could lead to a more rapid deterioration of patients with cirrhosis and hepatocellular carcinoma^29–32^. Interestingly, a recent investigation using GBD 2019 data found that Egypt had the highest incidence of acute viral hepatitis among the MENA countries^33^. The present study found that Egypt had the highest mortality rate due to alcohol, possibly due to the synergic association between the pathophysiology of viral hepatitis and alcohol consumption. Another important factor, particularly for liver cirrhosis, is the quality of the alcoholic drinks^34–36^. Counterproductive alcohol bans, which have been applied as a result of religious beliefs, may encourage the consumption of illegally produced and smuggled alcoholic drinks^37^. This may help to explain the relatively high burden of alcohol attributable digestive diseases in the MENA region.

A causal relationship between alcohol consumption and the development of multiple types of cancer has long been debated. Using the 2020 GLOBOCAN data, it was estimated that globally nearly 4.1% of all new cancer cases were alcohol-related^38^. Furthermore, given the increased aging of the population, alcohol related disability and death is predicted to rise in the near future, particularly because neoplasms disproportionately affect older adults. Cancers of the esophagus, pharynx, lip and oral cavity had the highest PAFs due to alcohol^38^. The incidence of these cancers have been linked to direct contact with acetaldehyde, an immediate metabolite of ethanol^39^. Acetaldehyde is responsible for some of the alcohol related carcinogenicity, via interference with the DNA repair process and damage to the DNA strands within the cells^40^. Apart from cancers of the upper aerodigestive tract, there have been reports that even a small intake of alcohol could substantially increase the risk of breast cancer among females^41^, which may be due to impaired regulation of hormones, such as estrogens and androgens^42^.

It is important to note that most MENA countries have faced population growth and cultural transitions in recent years, so although alcohol consumption was low in MENA, this rate may have increased. Furthermore, there are a number of factors which may increase the consumption of alcoholic drinks among these countries. For instance, by considering alcohol consumption to be a westernized, open-minded, and modern behavior, many young people in developing countries are attracted to drinking alcohol, with little knowledge about the likely health outcomes. Furthermore, extended media coverage over the last few decades has gradually introduced different cultures and attitudes into Islamic countries, which has resulted in the promotion and normalization of alcohol consumption, particularly among the younger age groups. Moreover, alcohol suppliers and distributors have developed new strategies to circumvent the bans, with the aim of advertising well-known alcohol brands and increasing consumption of these alcoholic drinks. Consequently, the problem of alcohol consumption must be addressed in MENA countries as a public health issue, rather than just due to religious concern^43^.

Public awareness of the causal relationship between alcohol consumption and the risk of several diseases is low. Therefore, adding cancer and digestive disease warnings to alcoholic drink labels, in the same way as on cigarette packages, may help to discourage people from purchasing and consuming alcoholic drinks^44^ and may also help to increase public cooperation with alcohol control policies^45^. Due to the substantial burden caused by alcohol consumption, several international initiatives have been proposed. The WHO has endorsed a cost-effective and feasible global strategy of so-called “best buys” to reduce the harmful impact of alcohol consumption at the population level. The program involves policies to increase taxation and ban alcohol advertising^46^. Reducing the harmful use of alcohol has also been targeted by the Non-Communicable Diseases Global Monitoring Framework and the Sustainable Development Goals programs^47^. In addition to the implementation of control measures, monitoring and surveillance frameworks are required to track alcohol consumption trends and its related health consequences. With a surveillance system in place, decision makers would be more able to appropriately prioritise and amend policies and interventions, based on the current situation within the community. However, very few countries have well-developed systems which reliably record and monitor the morbidity and mortality attributable to alcohol consumption^5^.

Despite the observed decrease in the burden attributable to alcohol consumption, it is important that government agencies and other health care organisations implement policies to reduce alcohol consumption, in order to decrease the level of alcohol induced harm. Tailoring these interventions to religious countries is crucial, since although the religious-driven policy may restrict alcohol intake to a low level, it is not sufficient on its own. The integration of scientific and evidence-based alcohol control guidelines are needed within the health systems of the MENA countries.

## Limitations

The present study has several limitations, which must be taken into account when interpreting the findings. Firstly, the results were not stratified according to the individuals’ level of alcohol intake or according to their drinking status (drinking or abstention). Furthermore, the pattern of alcohol consumption within the last 12 months is an important predictor of risk^48^, but the estimates assumed a consistent drinking pattern over time, as the currently available information was not detailed enough. Moreover, in addition to the interconnected relationship between alcohol consumption and dependence on tobacco, it is now clear that there is a synergic carcinogenic relationship between the two for most neoplasms of the upper aerodigestive tract^49–51^. Therefore, it could be hypothesised that some of the alcohol-attributable burden may have been caused by tobacco use. Fourthly, our findings are susceptible to several biases, due to the lack of high quality prospective cohort studies and the possibility that there are a substantial number of unrecorded alcohol related diseases in low and middle income countries. In a number of countries, alcohol consumption may be greatly under-reported, due to sociocultural factors, such as stigma^52^. Fifthly, the estimates were calculated using alcohol sales data, meaning that the burden due to alcohol consumption may have been underestimated, as some illicit production is likely to have been missed. Finally, data sparseness on the consumption of alcohol among adolescents may introduce further biases, suggesting more investigations are needed to study youth drinking behavior and the harm caused by alcohol consumption in this age group. Taken together, these limitations indicate that the burden attributable to alcohol consumption may have been underestimated.

## Conclusions

In summary, alcohol consumption remains a leading risk factor for adverse health outcomes, and its relative importance is increasing. The consumption of alcohol is low in the MENA region and the disease burden attributable to alcohol consumption has decreased over the last three decades. Nevertheless, increasing public awareness about the risk of alcohol consumption, together with country-specific policies for marketing bans and restricting alcohol industry lobbying, particularly in rapidly developing countries, might help reduce alcohol consumption and its attributable burden. Further research is needed to investigate the effects of alcohol consumption patterns on the burden of different diseases, especially among adolescents. Moreover, providing data at the subnational level, and by residential area, may help health policymakers and should be considered in future research.

## Supplementary Information

Supplementary Figure S1.

Supplementary Figure S2.

Supplementary Figure S3.

Supplementary Figure S4.

Supplementary Legends.

Supplementary Table S1.

Supplementary Table S2.

Supplementary Table S3.

Supplementary Table S4.

## Data Availability

The data used for these analyses are all publicly available at http://ghdx.healthdata.org/gbd-results-tool.
