# Supplementary material for: Burden of diseases and injuries attributable to alcohol consumption in the Middle East and North Africa region, 1990–2019
Source: Sci Rep. 2022 Nov 11;12:19301. doi: 10.1038/s41598-022-22901-x (PMC9652338; doi:10.1038/s41598-022-22901-x)
Supplement: Supplementary file 2 — Supplementary Figure S2. [file 41598_2022_22901_MOESM2_ESM.pdf]

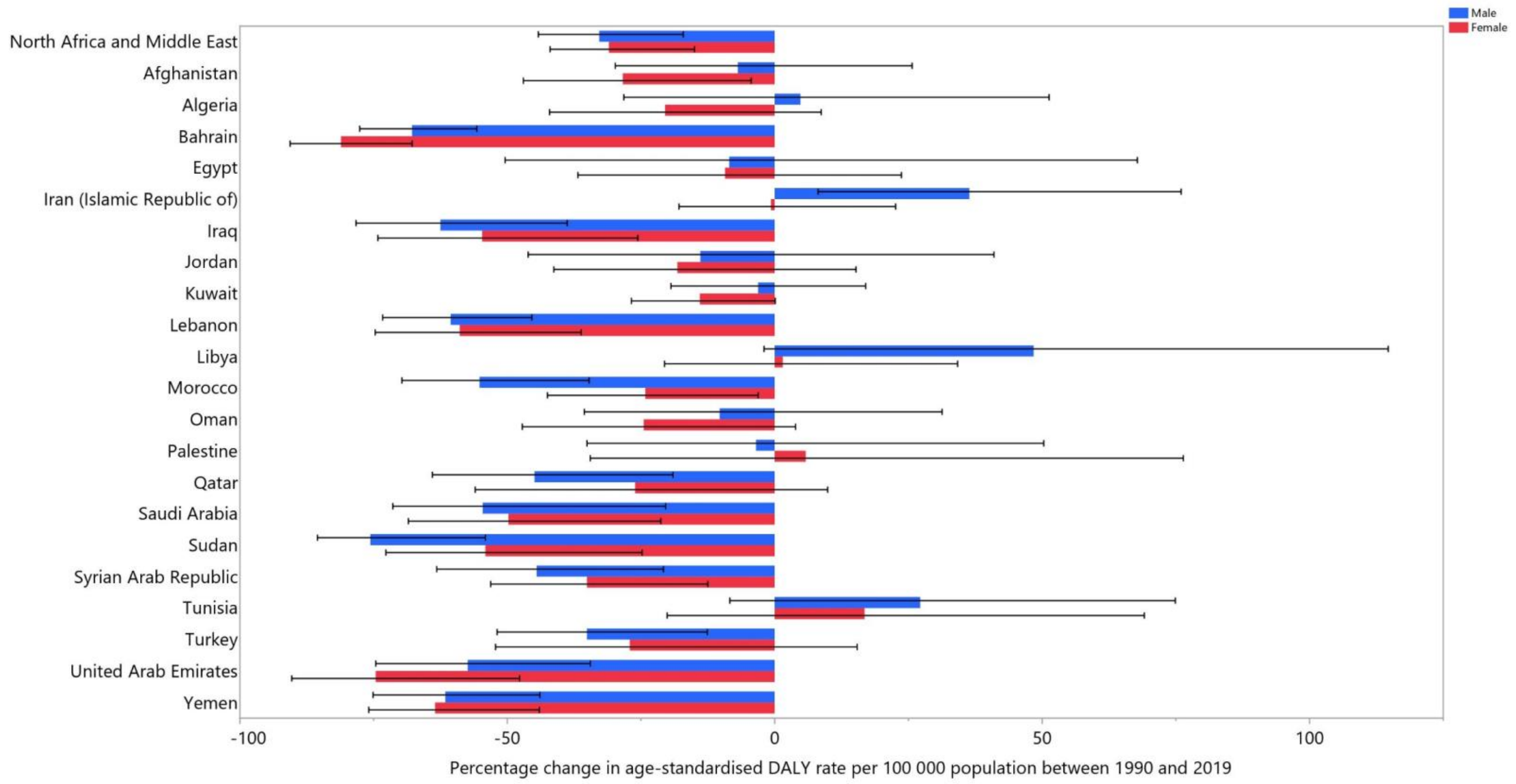

Figure S2: The percentage in the age-standardised DALYs rate of disease and injuries attributable to alcohol consumption in the Middle East and North Africa region from 1990 to 2019, by sex and country. DALY=disability-adjusted-life-years. (Generated from data available from http://ghdx.healthdata.org/gbd-results-tool).
