# Supplementary figures and images for: Burden of diseases and injuries attributable to alcohol consumption in the Middle East and North Africa region, 1990–2019
Source: Sci Rep. 2022 Nov 11;12:19301. doi: 10.1038/s41598-022-22901-x (PMC9652338; doi:10.1038/s41598-022-22901-x)

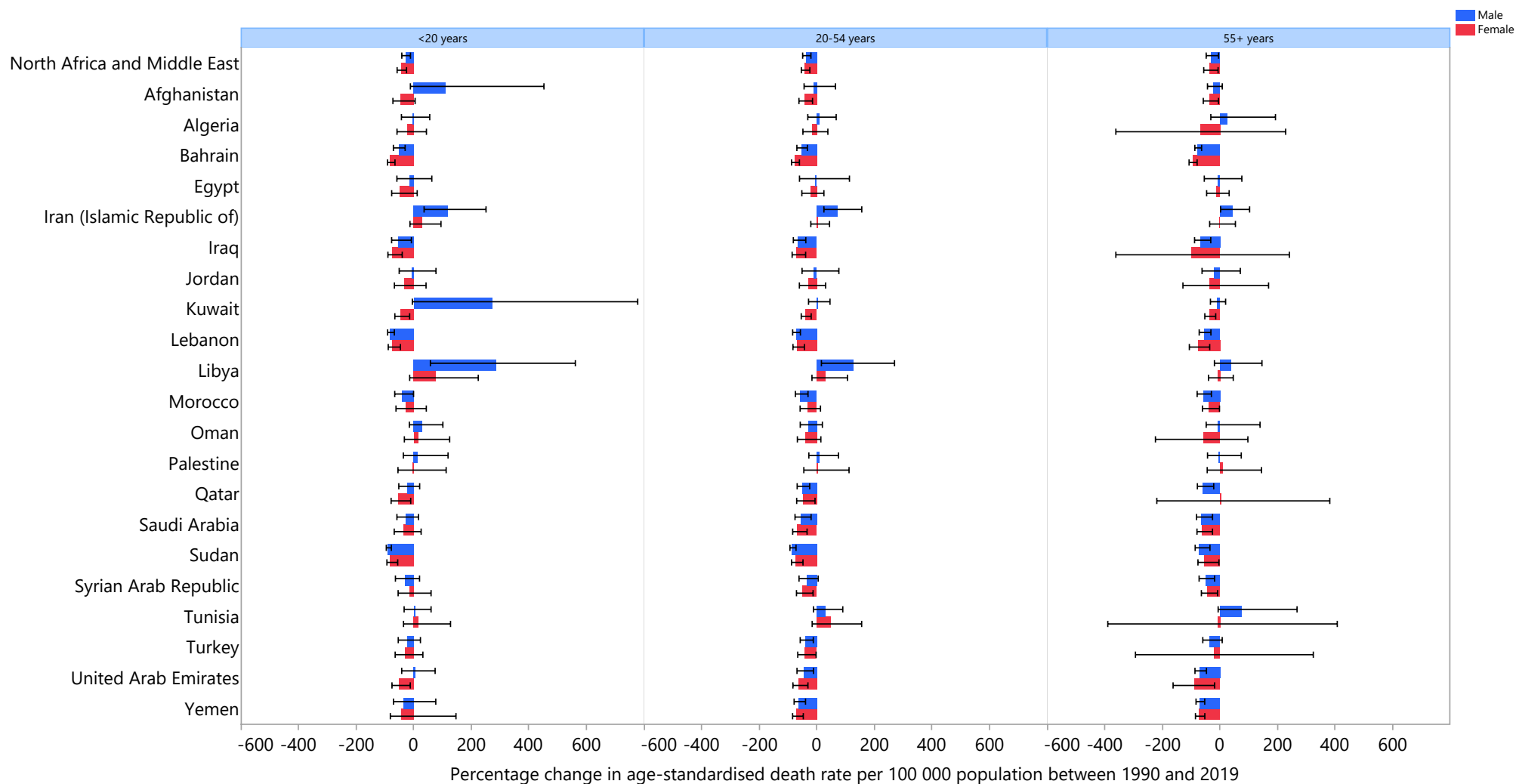

Supplement: Supplementary file 3 — Supplementary Figure S3. [file 41598_2022_22901_MOESM3_ESM.pdf]

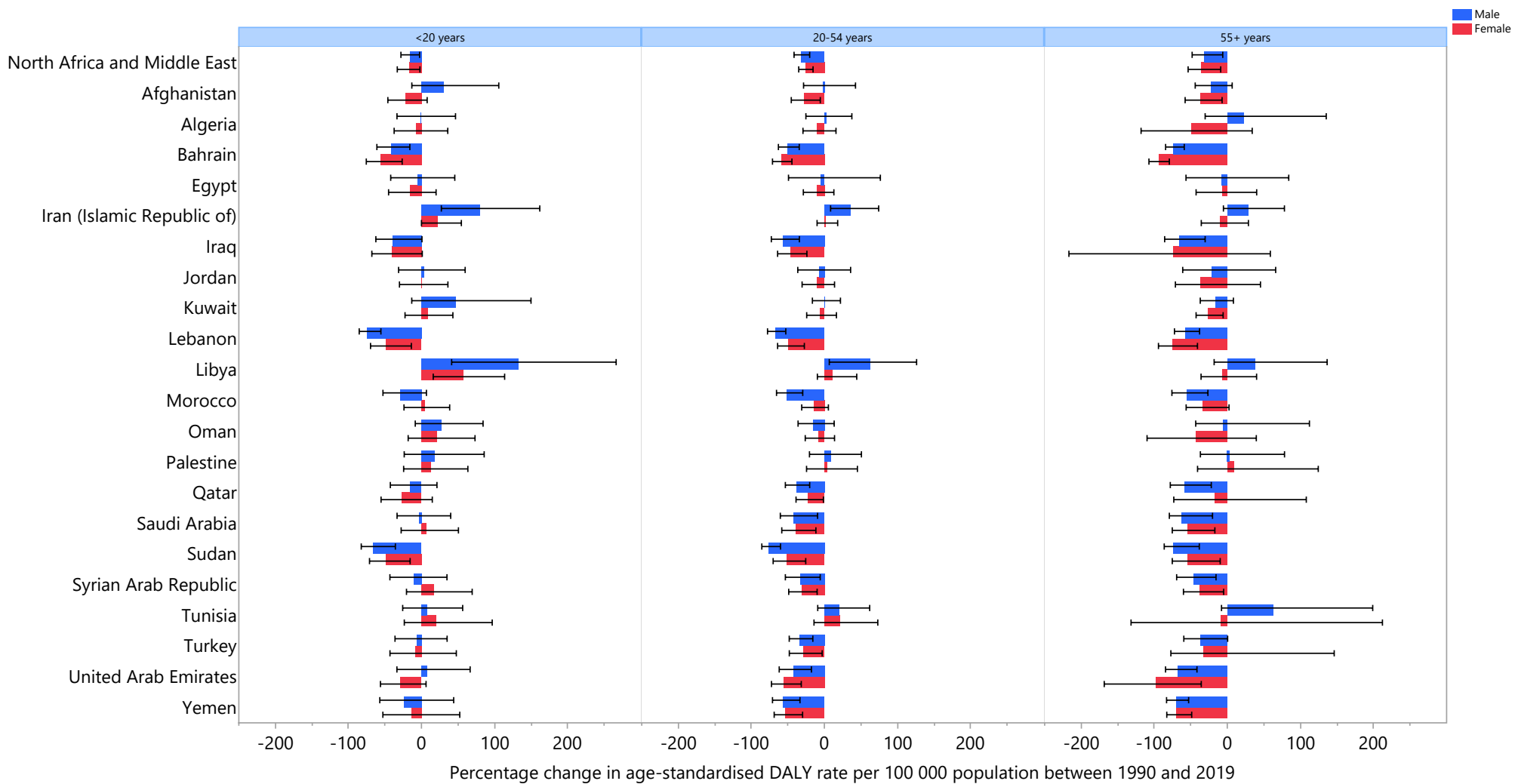

Supplement: Supplementary file 4 — Supplementary Figure S4. [file 41598_2022_22901_MOESM4_ESM.pdf]
