# Supplementary material for: Burden of diseases and injuries attributable to alcohol consumption in the Middle East and North Africa region, 1990–2019
Source: Sci Rep. 2022 Nov 11;12:19301. doi: 10.1038/s41598-022-22901-x (PMC9652338; doi:10.1038/s41598-022-22901-x)
Supplement: Supplementary file 5 — Supplementary Legends. [file 41598_2022_22901_MOESM5_ESM.docx]

**Supplementary table and figure legends**

**Table S1:** Deaths attributable to alcohol consumption in the Middle East and North Africa region in 2019 by sex (Generated from data available from <http://ghdx.healthdata.org/gbd-results-tool>).

**Table S2:** Deaths attributable to alcohol consumption in the Middle East and North Africa region in 1990 and 2019 (Generated from data available from <http://ghdx.healthdata.org/gbd-results-tool>).

**Table S3:** DALYs attributable to alcohol consumption in the Middle East and North Africa region in 2019 by sex. DALY=disability-adjusted-life-years. (Generated from data available from <http://ghdx.healthdata.org/gbd-results-tool>).

**Table S4:** DALYs attributable to alcohol use in the Middle East and North Africa region in 1990 and 2019. DALY=disability-adjusted-life-years. (Generated from data available from <http://ghdx.healthdata.org/gbd-results-tool>).

**Figure S1**: The age-standardised DALYs rate of disease and injuries attributable to alcohol consumption in the Middle East and North Africa region, by sex and country. DALY=disability-adjusted-life-years. (Generated from data available from <http://ghdx.healthdata.org/gbd-results-tool>).

**Figure S2**: The percentage in the age-standardised DALYs rate of disease and injuries attributable to alcohol consumption in the Middle East and North Africa region from 1990 to 2019, by sex and country. DALY=disability-adjusted-life-years. (Generated from data available from <http://ghdx.healthdata.org/gbd-results-tool>).

**Figure S3:** The percentage in the age-standardised death rate of disease and injuries attributable to alcohol consumption in the Middle East and North Africa region from 1990 to 2019 in <20, 20-54 and ≥55 age groups, by sex. (Generated from data available from <http://ghdx.healthdata.org/gbd-results-tool>).

**Figure S4:** The percentage in the age-standardised DALY rate of disease and injuries attributable to alcohol consumption in the Middle East and North Africa region from 1990 to 2019 in <20, 20-54 and ≥55 age groups, by sex. DALY=disability-adjusted-life-years. (Generated from data available from <http://ghdx.healthdata.org/gbd-results-tool>).
