# Supplementary material for: Burden of diseases and injuries attributable to alcohol consumption in the Middle East and North Africa region, 1990–2019
Source: Sci Rep. 2022 Nov 11;12:19301. doi: 10.1038/s41598-022-22901-x (PMC9652338; doi:10.1038/s41598-022-22901-x)
Supplement: Supplementary file 6 — Supplementary Table S1. [file 41598_2022_22901_MOESM6_ESM.doc]

| **Table S1: Deaths attributable to alcohol use in the Middle East and North Africa region in 2019 by sex**  **(Generated from data available from http://ghdx.healthdata.org/gbd-results-tool)** | | | | | | | | | | |
| --- | --- | --- | --- | --- | --- | --- | --- | --- | --- | --- |
|  | **Male** | | | | | **Female** | | | | |
|  | **No**  **(95% UI)** | **PAF**  **(95% UI)** | **ASRs per 100,000 (95% UI)** | **% change in ASRs per 100,000**  **1990-2019** | **Average annual percentage change (95% UI)** | **No**  **(95% UI)** | **PAF**  **(95% UI)** | **ASRs per 100,000 (95% UI)** | **% change in ASRs per 100,000**  **1990-2019** | **Average annual % change**  **1990-2019** |
| **North Africa and Middle East** | **18743 (13677 , 25389)** | **1.1 (0.8 , 1.4)** | **7.7 (5.6 , 10.5)** | **-34.2 (-48.6 , -12.2)** | **-1.43 (-1.76 , -1.10)** | **3237 (2121 , 4752)** | **0.2 (0.2 , 0.3)** | **1.5 (0.9 , 2.2)** | **-39.6 (-57 , -13.1)** | **-1.72 (-1.82 , -1.61)** |
| **Afghanistan** | **401 (253 , 601)** | **0.3 (0.2 , 0.4)** | **5.2 (3.3 , 7.4)** | **-13.5 (-37.6 , 21)** | **-0.48 (-0.64 , -0.33)** | **182 (108 , 270)** | **0.1 (0.1 , 0.2)** | **2.5 (1.4 , 3.8)** | **-34.9 (-55.8 , -5.5)** | **-1.41 (-1.50 , -1.32)** |
| **Algeria** | **1203 (719 , 1857)** | **1.1 (0.7 , 1.7)** | **6.3 (3.5 , 9.9)** | **11.4 (-33.9 , 100.6)** | **0.38 (0.22 , 0.55)** | **82 (4 , 173)** | **0.1 (0 , 0.2)** | **0.2 (-0.5 , 0.9)** | **-64.3 (-348.3 , 199.8)** | **-3.49 (-3.60 , -3.38)** |
| **Bahrain** | **66 (42 , 97)** | **2.5 (1.7 , 3.6)** | **8.6 (5.2 , 13.3)** | **-75.8 (-85.2 , -62.9)** | **-4.90 (-5.50 , -4.29)** | **2 (0 , 5)** | **0.1 (0 , 0.3)** | **0.3 (-0.5 , 1.1)** | **-95.7 (-112.5 , -80.9)** | **-10.30 (-10.82 , -9.78)** |
| **Egypt** | **5064 (2653 , 8880)** | **1.6 (0.9 , 2.6)** | **14.4 (7.7 , 24.2)** | **-9.3 (-54 , 67.7)** | **-0.37 (-0.89 , 0.15)** | **1143 (576 , 2010)** | **0.5 (0.3 , 0.8)** | **5.2 (2.7 , 8.9)** | **-6.1 (-40.6 , 34.3)** | **-0.19 (-0.56 , 0.19)** |
| **Iran (Islamic Republic of)** | **2560 (1922 , 3433)** | **1.1 (0.9 , 1.5)** | **6.5 (4.8 , 8.8)** | **49.5 (10.9 , 106.7)** | **1.42 (1.21 , 1.63)** | **416 (302 , 585)** | **0.2 (0.2 , 0.3)** | **1.2 (0.8 , 1.7)** | **-2.8 (-34.7 , 49.7)** | **-0.04 (-0.32 , 0.24)** |
| **Iraq** | **778 (394 , 1358)** | **0.7 (0.4 , 1.2)** | **5 (2.3 , 9)** | **-69.4 (-86.1 , -42)** | **-4.00 (-4.25 , -3.74)** | **39 (-11 , 99)** | **0.1 (0 , 0.1)** | **0.1 (-0.4 , 0.6)** | **-89.8 (-267.4 , 21.3)** | **-7.49 (-8.39 , -6.58)** |
| **Jordan** | **171 (86 , 283)** | **0.9 (0.5 , 1.4)** | **4 (2 , 6.8)** | **-17.2 (-57.4 , 65.1)** | **-0.60 (-0.91 , -0.29)** | **15 (6 , 29)** | **0.1 (0 , 0.2)** | **0.4 (0 , 0.9)** | **-35.9 (-114.3 , 154.6)** | **-1.48 (-1.75 , -1.22)** |
| **Kuwait** | **28 (20 , 40)** | **0.4 (0.3 , 0.5)** | **1.6 (1.1 , 2.3)** | **-5.5 (-29.5 , 23.1)** | **-0.18 (-1.15 , 0.80)** | **4 (3 , 6)** | **0.1 (0.1 , 0.2)** | **0.4 (0.2 , 0.5)** | **-39.3 (-54.6 , -19.3)** | **-1.68 (-2.69 , -0.66)** |
| **Lebanon** | **239 (135 , 374)** | **1.3 (0.7 , 1.9)** | **10.1 (5.7 , 15.8)** | **-60.4 (-75.8 , -41.7)** | **-3.00 (-3.23 , -2.78)** | **24 (3 , 54)** | **0.2 (0 , 0.4)** | **0.8 (0.1 , 1.9)** | **-75.3 (-95.9 , -41.8)** | **-4.69 (-4.85 , -4.53)** |
| **Libya** | **175 (101 , 265)** | **1 (0.6 , 1.4)** | **5.8 (3.4 , 8.8)** | **54.8 (-9.5 , 158.6)** | **1.52 (0.79 , 2.26)** | **34 (23 , 47)** | **0.2 (0.2 , 0.3)** | **1.3 (0.9 , 1.9)** | **-5.2 (-37.4 , 45)** | **-0.14 (-0.60 , 0.32)** |
| **Morocco** | **953 (501 , 1516)** | **0.8 (0.4 , 1.2)** | **5.4 (2.8 , 8.7)** | **-61.2 (-77 , -38.4)** | **-3.23 (-3.47 , -3.00)** | **155 (105 , 220)** | **0.1 (0.1 , 0.2)** | **1 (0.7 , 1.4)** | **-38.5 (-60.4 , -5)** | **-1.65 (-1.70 , -1.59)** |
| **Oman** | **63 (31 , 103)** | **0.8 (0.4 , 1.3)** | **4 (1.8 , 6.5)** | **-9.9 (-44.4 , 68.7)** | **-0.42 (-0.87 , 0.03)** | **3 (0 , 7)** | **0.1 (0 , 0.2)** | **0.2 (-0.4 , 0.8)** | **-68.4 (-254.2 , 87.5)** | **-3.86 (-5.04 , -2.67)** |
| **Palestine** | **179 (130 , 240)** | **2 (1.5 , 2.6)** | **14.6 (10.1 , 20)** | **-2.5 (-40 , 69.7)** | **-0.12 (-0.52 , 0.27)** | **41 (23 , 67)** | **0.5 (0.3 , 0.9)** | **3.5 (1.9 , 5.9)** | **10 (-43.6 , 139.4)** | **0.33 (0.10 , 0.57)** |
| **Qatar** | **73 (47 , 105)** | **2.2 (1.5 , 2.9)** | **8.5 (5.4 , 12.4)** | **-42.2 (-65.1 , -5.9)** | **-1.97 (-3.04 , -0.88)** | **3 (1 , 5)** | **0.3 (0.1 , 0.4)** | **1 (-0.4 , 2.4)** | **-18 (-225.1 , 248.5)** | **-0.60 (-1.21 , 0.01)** |
| **Saudi Arabia** | **641 (293 , 1276)** | **0.7 (0.4 , 1.4)** | **4.8 (2.4 , 9.1)** | **-62.3 (-78.3 , -23.9)** | **-3.35 (-3.67 , -3.03)** | **80 (45 , 136)** | **0.2 (0.1 , 0.3)** | **1.2 (0.6 , 2)** | **-62.1 (-80.2 , -27)** | **-3.27 (-3.43 , -3.11)** |
| **Sudan** | **379 (218 , 607)** | **0.3 (0.2 , 0.5)** | **3.7 (2.1 , 6)** | **-77.7 (-88.5 , -49.9)** | **-5.11 (-5.53 , -4.68)** | **115 (69 , 177)** | **0.1 (0.1 , 0.2)** | **1.3 (0.8 , 2.1)** | **-60.4 (-79.3 , -21.1)** | **-3.10 (-3.40 , -2.78)** |
| **Syrian Arab Republic** | **490 (252 , 840)** | **1 (0.6 , 1.6)** | **7.7 (3.9 , 13.1)** | **-49.7 (-71.7 , -21.3)** | **-2.40 (-2.75 , -2.04)** | **78 (49 , 115)** | **0.2 (0.1 , 0.3)** | **1.5 (0.9 , 2.1)** | **-42.3 (-63.3 , -10.6)** | **-1.95 (-2.23 , -1.66)** |
| **Tunisia** | **645 (400 , 977)** | **1.7 (1.2 , 2.3)** | **10.4 (6.3 , 15.8)** | **44.2 (-10.6 , 130.9)** | **1.28 (1.10 , 1.46)** | **78 (29 , 144)** | **0.3 (0.1 , 0.4)** | **1.2 (0.4 , 2.2)** | **19.7 (-49.6 , 215.8)** | **0.64 (0.43 , 0.86)** |
| **Turkey** | **3343 (2257 , 4680)** | **1.4 (1 , 1.8)** | **7.6 (5.1 , 10.6)** | **-36 (-58.4 , -1.6)** | **-1.52 (-1.79 , -1.24)** | **598 (183 , 1138)** | **0.3 (0.1 , 0.5)** | **1.3 (0.4 , 2.4)** | **-27.6 (-77.7 , 130.9)** | **-1.25 (-1.79 , -0.72)** |
| **United Arab Emirates** | **535 (297 , 854)** | **2.2 (1.3 , 3.3)** | **11.6 (5.6 , 18.7)** | **-66.2 (-82.8 , -43.7)** | **-3.72 (-4.03 , -3.40)** | **17 (6 , 33)** | **0.3 (0.1 , 0.6)** | **0.8 (-0.4 , 2.2)** | **-81.1 (-124.9 , -29.4)** | **NA** |
| **Yemen** | **738 (416 , 1138)** | **0.7 (0.4 , 1.1)** | **8.9 (4.9 , 13.9)** | **-67.8 (-80.7 , -50.1)** | **-3.82 (-3.96 , -3.68)** | **123 (75 , 198)** | **0.2 (0.1 , 0.2)** | **1.7 (1 , 2.7)** | **-73.4 (-84.6 , -53.9)** | **-4.45 (-4.55 , -4.34)** |
