# Supplementary material for: Burden of diseases and injuries attributable to alcohol consumption in the Middle East and North Africa region, 1990–2019
Source: Sci Rep. 2022 Nov 11;12:19301. doi: 10.1038/s41598-022-22901-x (PMC9652338; doi:10.1038/s41598-022-22901-x)
Supplement: Supplementary file 7 — Supplementary Table S2. [file 41598_2022_22901_MOESM7_ESM.doc]

| **Table S2: Deaths attributable to alcohol use in the Middle East and North Africa region in 1990 and 2019**  **(Generated from data available from http://ghdx.healthdata.org/gbd-results-tool)** | | | | | | | | |
| --- | --- | --- | --- | --- | --- | --- | --- | --- |
|  | **1990** | | | **2019** | | | **% change in ASRs per 100,000**  **1990-2019** | **Average annual % change 1990-2019** |
|  | **No**  **(95% UI)** | **PAF**  **(95% UI)** | **ASRs per 100,000 (95% UI)** | **No**  **(95% UI)** | **PAF**  **(95% UI)** | **ASRs per 100,000 (95% UI)** |
| **North Africa and Middle East** | **13918 (10508 , 17820)** | **0.5 (0.4 , 0.7)** | **7.1 (5.3 , 9.1)** | **21980 (16085 , 29408)** | **0.7 (0.5 , 0.9)** | **4.6 (3.4 , 6.3)** | **-34.5 (-48.3 , -13.2)** | **-1.45 (-1.78 , -1.12)** |
| **Afghanistan** | **356 (220 , 513)** | **0.2 (0.1 , 0.3)** | **5 (3.2 , 7)** | **583 (375 , 853)** | **0.2 (0.2 , 0.3)** | **3.8 (2.5 , 5.4)** | **-23.1 (-43.9 , 4.2)** | **-0.86 (-1.07 , -0.65)** |
| **Algeria** | **496 (295 , 727)** | **0.3 (0.2 , 0.5)** | **3.2 (1.6 , 5)** | **1285 (766 , 1972)** | **0.6 (0.4 , 1)** | **3.3 (1.9 , 5.3)** | **5.8 (-36.8 , 82.3)** | **0.23 (0.09 , 0.37)** |
| **Bahrain** | **49 (37 , 62)** | **2.5 (2 , 3.1)** | **21.5 (15.5 , 28.7)** | **69 (44 , 101)** | **1.6 (1.1 , 2.3)** | **5.1 (3 , 8)** | **-76.2 (-85.7 , -62.3)** | **-4.96 (-5.59 , -4.33)** |
| **Egypt** | **3042 (1832 , 4619)** | **0.7 (0.4 , 1)** | **10.7 (6.6 , 16.3)** | **6207 (3373 , 10443)** | **1.1 (0.6 , 1.8)** | **10.1 (5.7 , 16.6)** | **-5.6 (-46.7 , 57.4)** | **-0.21 (-.0.64 , 0.23)** |
| **Iran (Islamic Republic of)** | **837 (627 , 1138)** | **0.2 (0.2 , 0.3)** | **2.8 (2.1 , 3.7)** | **2976 (2244 , 3996)** | **0.8 (0.6 , 1)** | **3.8 (2.9 , 5.2)** | **36.7 (2.7 , 89.6)** | **1.10 (0.93 , 1.27)** |
| **Iraq** | **831 (505 , 1221)** | **0.7 (0.4 , 1)** | **8.6 (5 , 13.1)** | **817 (406 , 1418)** | **0.5 (0.2 , 0.8)** | **2.5 (1.1 , 4.6)** | **-70.6 (-87.2 , -42.9)** | **-4.13 (-4.39 , -3.86)** |
| **Jordan** | **50 (25 , 87)** | **0.3 (0.2 , 0.6)** | **2.8 (1.4 , 5.1)** | **186 (97 , 309)** | **0.6 (0.3 , 0.9)** | **2.3 (1.2 , 4)** | **-16.5 (-56.6 , 65.5)** | **-0.58 (-0.85 , -0.30)** |
| **Kuwait** | **10 (8 , 12)** | **0.2 (0.2 , 0.2)** | **1.3 (1 , 1.6)** | **32 (23 , 45)** | **0.3 (0.2 , 0.4)** | **1.1 (0.8 , 1.5)** | **-14.5 (-34.1 , 8.9)** | **-0.51 (-1.56 , 0.55)** |
| **Lebanon** | **341 (247 , 457)** | **1.5 (1.1 , 2)** | **14.1 (10.1 , 19.1)** | **262 (147 , 419)** | **0.8 (0.5 , 1.2)** | **5 (2.8 , 8)** | **-64.3 (-78.3 , -46.1)** | **-3.39 (-3.59 , -3.19)** |
| **Libya** | **54 (37 , 76)** | **0.3 (0.2 , 0.4)** | **2.6 (1.7 , 3.9)** | **209 (124 , 310)** | **0.7 (0.4 , 0.9)** | **3.6 (2.2 , 5.4)** | **35.9 (-15.4 , 120.4)** | **1.25 (0.52 , 1.98)** |
| **Morocco** | **1275 (895 , 1723)** | **0.7 (0.5 , 0.9)** | **7.8 (5.2 , 10.7)** | **1108 (638 , 1698)** | **0.5 (0.3 , 0.7)** | **3.2 (1.8 , 5)** | **-58.4 (-73.8 , -36.7)** | **-3.00 (-3.22 , -2.78)** |
| **Oman** | **27 (13 , 45)** | **0.3 (0.1 , 0.4)** | **2.6 (1 , 4.5)** | **67 (33 , 108)** | **0.5 (0.3 , 0.9)** | **2.3 (1 , 3.9)** | **-12.6 (-47 , 62.1)** | **-0.49 (-1.00 , 0.02)** |
| **Palestine** | **78 (43 , 125)** | **0.6 (0.4 , 1)** | **8.5 (4.5 , 13.7)** | **219 (155 , 298)** | **1.3 (1 , 1.7)** | **8.6 (5.8 , 12.2)** | **1.9 (-37.9 , 85.6)** | **0.09 (-0.14 , 0.32)** |
| **Qatar** | **18 (12 , 25)** | **1.3 (0.9 , 1.9)** | **9.2 (5.8 , 13.6)** | **75 (49 , 110)** | **1.7 (1.2 , 2.3)** | **6.8 (4.2 , 10)** | **-26.4 (-56 , 19.7)** | **-1.09 (-2.32 , 0.15)** |
| **Saudi Arabia** | **609 (210 , 1154)** | **0.7 (0.3 , 1.4)** | **8.8 (3.1 , 17.4)** | **722 (352 , 1405)** | **0.6 (0.3 , 1)** | **3.4 (1.8 , 6.1)** | **-61.9 (-77.7 , -26.4)** | **-3.32 (-3.55 , -3.08)** |
| **Sudan** | **1067 (467 , 1745)** | **0.4 (0.2 , 0.7)** | **10.1 (4.3 , 16.9)** | **494 (300 , 774)** | **0.2 (0.2 , 0.4)** | **2.6 (1.5 , 4)** | **-74.4 (-86.5 , -44.6)** | **-4.63 (-4.98 , -4.28)** |
| **Syrian Arab Republic** | **533 (353 , 769)** | **0.7 (0.5 , 1)** | **9.2 (6 , 13.5)** | **567 (306 , 938)** | **0.7 (0.4 , 1)** | **4.6 (2.5 , 7.7)** | **-49.5 (-70.1 , -22)** | **-2.37 (-2.71 , -2.03)** |
| **Tunisia** | **264 (177 , 368)** | **0.6 (0.4 , 0.8)** | **4.1 (2.5 , 5.9)** | **723 (446 , 1109)** | **1.1 (0.7 , 1.5)** | **5.6 (3.4 , 8.6)** | **37.1 (-15.9 , 120.2)** | **1.11 (0.96 , 1.26)** |
| **Turkey** | **2889 (1933 , 4013)** | **0.7 (0.5 , 1)** | **6.7 (4.3 , 9.5)** | **3941 (2551 , 5734)** | **0.9 (0.6 , 1.2)** | **4.3 (2.7 , 6.4)** | **-35.6 (-58.8 , 1.7)** | **-1.56 (-1.83 , -1.28)** |
| **United Arab Emirates** | **164 (115 , 222)** | **2.8 (2.1 , 3.7)** | **22.8 (14.4 , 33)** | **553 (306 , 884)** | **1.9 (1.1 , 2.8)** | **8.4 (4 , 13.8)** | **-63.1 (-81.4 , -37.5)** | **-3.41 (-3.82 , -3.00)** |
| **Yemen** | **920 (591 , 1336)** | **0.6 (0.4 , 0.9)** | **16.2 (10.5 , 23.9)** | **861 (492 , 1325)** | **0.5 (0.3 , 0.7)** | **5.2 (2.9 , 8.1)** | **-67.9 (-80.1 , -50.2)** | **-3.84 (-3.97 , -3.70)** |
