# Supplementary material for: Burden of diseases and injuries attributable to alcohol consumption in the Middle East and North Africa region, 1990–2019
Source: Sci Rep. 2022 Nov 11;12:19301. doi: 10.1038/s41598-022-22901-x (PMC9652338; doi:10.1038/s41598-022-22901-x)
Supplement: Supplementary file 8 — Supplementary Table S3. [file 41598_2022_22901_MOESM8_ESM.doc]

| **Table S3: DALYs attributable to alcohol use in the Middle East and North Africa region in 2019 by sex**  **(Generated from data available from http://ghdx.healthdata.org/gbd-results-tool)** | | | | | | | | | | |
| --- | --- | --- | --- | --- | --- | --- | --- | --- | --- | --- |
|  | **Male** | | | | | **Female** | | | | |
|  | **No**  **(95% UI)** | **PAF**  **(95% UI)** | **ASRs per 100,000 (95% UI)** | **% change in ASRs per 100,000**  **1990-2019** | **Average annual percentage change (95% UI)** | **No**  **(95% UI)** | **PAF**  **(95% UI)** | **ASRs per 100,000 (95% UI)** | **% change in ASRs per 100,000**  **1990-2019** | **Average annual % change**  **1990-2019** |
| **North Africa and Middle East** | **877973 (691407 , 1104821)** | **1 (0.8 , 1.2)** | **301.1 (235.6 , 385.3)** | **-32.8 (-44.2 , -17.1)** | **-1.35 (-1.51 , -1.19)** | **181818 (138567 , 232008)** | **0.2 (0.2 , 0.3)** | **67 (50.5 , 86.4)** | **-31 (-42 , -15)** | **-1.28 (-1.37 , -1.19)** |
| **Afghanistan** | **24954 (17313 , 34153)** | **0.3 (0.2 , 0.4)** | **215.8 (151.3 , 293.1)** | **-6.9 (-29.8 , 25.7)** | **-0.24 (-0.36 , -0.11)** | **10586 (7578 , 14167)** | **0.1 (0.1 , 0.2)** | **96.1 (65.7 , 132.2)** | **-28.4 (-47 , -4.4)** | **-1.11 (-1.18 , -1.05)** |
| **Algeria** | **62345 (44087 , 84918)** | **1.2 (0.9 , 1.6)** | **293.8 (204.7 , 402.8)** | **4.8 (-28.2 , 51.3)** | **0.17 (0.07 , 0.28)** | **9777 (6304 , 13774)** | **0.2 (0.1 , 0.3)** | **43.2 (25.6 , 62.6)** | **-20.5 (-42.1 , 8.7)** | **-0.78 (-0.92 , -0.65)** |
| **Bahrain** | **3593 (2572 , 4913)** | **2.1 (1.5 , 2.7)** | **347.7 (244.8 , 487.7)** | **-67.8 (-77.6 , -55.7)** | **-3.94 (-4.28 , -3.59)** | **266 (168 , 389)** | **0.2 (0.2 , 0.3)** | **38.4 (18.6 , 60.9)** | **-81.1 (-90.6 , -67.8)** | **-5.62 (-5.79 , -5.45)** |
| **Egypt** | **180796 (105680 , 302446)** | **1.3 (0.8 , 2)** | **438.5 (253.3 , 726.4)** | **-8.5 (-50.4 , 67.8)** | **-0.36 (-0.78 , 0.07)** | **40399 (26556 , 61047)** | **0.3 (0.2 , 0.5)** | **124.4 (75.9 , 196.9)** | **-9.3 (-36.8 , 23.7)** | **-0.28 (-0.58 , 0.03)** |
| **Iran (Islamic Republic of)** | **117223 (91263 , 148028)** | **1.1 (0.9 , 1.4)** | **263.3 (204.7 , 334.1)** | **36.4 (8.1 , 76)** | **1.09 (0.86 , 1.32)** | **22610 (17489 , 28833)** | **0.2 (0.2 , 0.3)** | **52.9 (41 , 67.9)** | **-0.7 (-17.9 , 22.6)** | **0.00 (-0.18 , 0.19)** |
| **Iraq** | **46834 (29327 , 72215)** | **0.8 (0.6 , 1.2)** | **247.4 (147.1 , 393.6)** | **-62.5 (-78.3 , -38.8)** | **-3.33 (-3.53 , -3.13)** | **7714 (4852 , 11121)** | **0.2 (0.1 , 0.2)** | **36 (20.3 , 54.1)** | **-54.7 (-74.2 , -25.6)** | **-2.70 (-2.79 , -2.61)** |
| **Jordan** | **10628 (6761 , 15119)** | **0.9 (0.6 , 1.3)** | **191.9 (117.8 , 284.8)** | **-13.9 (-46.1 , 41)** | **-0.49 (-0.74 , -0.24)** | **1988 (1319 , 2758)** | **0.2 (0.1 , 0.3)** | **38.8 (25.4 , 54.8)** | **-18.2 (-41.3 , 15.2)** | **-0.70 (-0.85 , -0.55)** |
| **Kuwait** | **2565 (1845 , 3477)** | **0.6 (0.5 , 0.8)** | **94.8 (69.7 , 125.1)** | **-3.1 (-19.4 , 17)** | **-0.10 (-0.66 , 0.45)** | **799 (520 , 1152)** | **0.2 (0.2 , 0.3)** | **34.2 (23.3 , 46.6)** | **-14 (-26.8 , 0.1)** | **-0.54 (-0.81 , -0.26)** |
| **Lebanon** | **9358 (6303 , 13341)** | **1.4 (0.9 , 1.9)** | **376.9 (253.2 , 542.7)** | **-60.6 (-73.3 , -45.4)** | **-2.97 (-3.19 , -2.75)** | **1478 (887 , 2253)** | **0.2 (0.1 , 0.3)** | **52.6 (32 , 80.3)** | **-58.9 (-74.7 , -36.2)** | **-3.01 (-3.09 , -2.93)** |
| **Libya** | **9148 (5770 , 12561)** | **1 (0.7 , 1.3)** | **250.2 (158.5 , 347.4)** | **48.4 (-2 , 114.7)** | **1.38 (0.80 , 1.95)** | **2037 (1508 , 2698)** | **0.3 (0.2 , 0.3)** | **61.7 (45.6 , 81.5)** | **1.5 (-20.6 , 34.2)** | **0.07 (-0.23 , 0.38)** |
| **Morocco** | **49182 (32104 , 71891)** | **1 (0.7 , 1.3)** | **263.5 (169.3 , 387)** | **-55.2 (-69.7 , -34.7)** | **-2.77 (-3.01 , -2.52)** | **9429 (6838 , 12452)** | **0.2 (0.1 , 0.2)** | **52.2 (38.2 , 68.3)** | **-24.2 (-42.5 , -3.1)** | **-0.95 (-1.01 , -0.89)** |
| **Oman** | **5433 (3557 , 7645)** | **1 (0.7 , 1.4)** | **181 (112 , 263.5)** | **-10.3 (-35.6 , 31.3)** | **-0.34 (-0.72 , 0.04)** | **624 (396 , 913)** | **0.2 (0.1 , 0.3)** | **36.4 (20.1 , 55.3)** | **-24.5 (-47.2 , 3.9)** | **-0.95 (-1.42 , -0.49)** |
| **Palestine** | **8046 (6184 , 10067)** | **1.5 (1.2 , 1.9)** | **470.8 (353 , 605.6)** | **-3.5 (-35.1 , 50.3)** | **-0.13 (-0.40 , 0.15)** | **1827 (1261 , 2548)** | **0.4 (0.3 , 0.5)** | **111.1 (73.3 , 162.3)** | **5.8 (-34.5 , 76.4)** | **0.16 (0.00 , 0.32)** |
| **Qatar** | **5394 (3875 , 7269)** | **1.6 (1.3 , 2.1)** | **272.1 (190.5 , 371)** | **-44.9 (-64 , -19)** | **-2.10 (-2.51 , -1.69)** | **321 (209 , 454)** | **0.3 (0.2 , 0.4)** | **47.5 (23.6 , 74)** | **-26.1 (-56 , 9.9)** | **-0.99 (-1.30 , -0.69)** |
| **Saudi Arabia** | **42920 (24680 , 70403)** | **0.8 (0.5 , 1.3)** | **199 (116.9 , 343.2)** | **-54.6 (-71.4 , -20.4)** | **-2.69 (-2.90 , -2.49)** | **7623 (4988 , 10883)** | **0.2 (0.2 , 0.3)** | **54.3 (36.2 , 76.4)** | **-49.8 (-68.5 , -21.3)** | **-2.32 (-2.47 , -2.18)** |
| **Sudan** | **21418 (15284 , 28846)** | **0.3 (0.2 , 0.4)** | **149.1 (103.9 , 207.1)** | **-75.6 (-85.5 , -54.1)** | **-4.86 (-5.46 , -4.26)** | **8345 (5928 , 11366)** | **0.1 (0.1 , 0.2)** | **57.6 (40.5 , 80.2)** | **-54.1 (-72.7 , -24.8)** | **-2.62 (-2.85 , -2.39)** |
| **Syrian Arab Republic** | **19733 (12106 , 30205)** | **1 (0.6 , 1.4)** | **290.5 (182 , 444.8)** | **-44.5 (-63.2 , -20.8)** | **-2.05 (-2.36 , -1.74)** | **4279 (3073 , 5630)** | **0.2 (0.2 , 0.3)** | **61.4 (44.3 , 80.6)** | **-35.1 (-53.1 , -12.5)** | **-1.50 (-1.76 , -1.24)** |
| **Tunisia** | **29188 (20628 , 39875)** | **1.9 (1.5 , 2.4)** | **464.6 (326 , 630.7)** | **27.2 (-8.4 , 74.9)** | **0.85 (0.66 , 1.05)** | **5426 (3474 , 7847)** | **0.4 (0.3 , 0.6)** | **84.4 (54.3 , 121.4)** | **16.8 (-20.1 , 69.1)** | **0.54 (0.45 , 0.63)** |
| **Turkey** | **157967 (117917 , 206689)** | **1.6 (1.2 , 1.9)** | **340.4 (253.7 , 447.8)** | **-35.1 (-51.9 , -12.6)** | **-1.53 (-1.80 , -1.27)** | **36571 (23332 , 52222)** | **0.4 (0.3 , 0.5)** | **79.4 (50.9 , 113.6)** | **-27.1 (-52.2 , 15.4)** | **-1.22 (-1.81 , -0.61)** |
| **United Arab Emirates** | **32528 (21360 , 47803)** | **2 (1.4 , 2.7)** | **465.3 (277.9 , 705.3)** | **-57.4 (-74.6 , -34.5)** | **-2.93 (-3.32 , -2.54)** | **1569 (934 , 2417)** | **0.3 (0.2 , 0.5)** | **47 (16.9 , 86.3)** | **-74.6 (-90.3 , -47.7)** | **-4.66 (-5.27 , -4.04)** |
| **Yemen** | **37829 (24548 , 54481)** | **0.6 (0.4 , 0.8)** | **352.4 (218.8 , 517.6)** | **-61.6 (-75.1 , -43.9)** | **-3.24 (-3.40 , -3.08)** | **7965 (5505 , 11119)** | **0.1 (0.1 , 0.2)** | **72.5 (49.7 , 105.9)** | **-63.5 (-75.9 , -44)** | **-3.42 (-3.54 , -3.31)** |
