# Supplementary material for: Burden of diseases and injuries attributable to alcohol consumption in the Middle East and North Africa region, 1990–2019
Source: Sci Rep. 2022 Nov 11;12:19301. doi: 10.1038/s41598-022-22901-x (PMC9652338; doi:10.1038/s41598-022-22901-x)
Supplement: Supplementary file 9 — Supplementary Table S4. [file 41598_2022_22901_MOESM9_ESM.doc]

| **Table S4: DALYs attributable to alcohol use in the Middle East and North Africa region in 1990 and 2019**  **(Generated from data available from http://ghdx.healthdata.org/gbd-results-tool)** | | | | | | | | |
| --- | --- | --- | --- | --- | --- | --- | --- | --- |
|  | **1990** | | | **2019** | | | **% change in ASRs per 100,000**  **1990-2019** | **Average annual % change 1990-2019** |
|  | **No**  **(95% UI)** | **PAF**  **(95% UI)** | **ASRs per 100,000 (95% UI)** | **No**  **(95% UI)** | **PAF**  **(95% UI)** | **ASRs per 100,000 (95% UI)** |  |  |
| **North Africa and Middle East** | **661856 (535026 , 804864)** | **0.4 (0.3 , 0.5)** | **276.6 (218.9 , 339)** | **1059791 (842123 , 1316847)** | **0.6 (0.5 , 0.8)** | **188.3 (148.5 , 235.7)** | **-31.9 (-42.5 , -16.9)** | **-1.31 (-1.46 , -1.15)** |
| **Afghanistan** | **14323 (9906 , 19512)** | **0.1 (0.1 , 0.2)** | **181.6 (127 , 245.8)** | **35540 (25680 , 47682)** | **0.2 (0.2 , 0.3)** | **155.6 (110.4 , 208.9)** | **-14.3 (-33.5 , 12.8)** | **-0.49 (-0.64 , -0.35)** |
| **Algeria** | **31823 (22497 , 42512)** | **0.3 (0.2 , 0.4)** | **167.2 (114.9 , 226.9)** | **72122 (51488 , 96915)** | **0.7 (0.5 , 0.9)** | **170.2 (119.7 , 231.3)** | **1.8 (-28.5 , 42.9)** | **0.06 (-0.03 , 0.14)** |
| **Bahrain** | **2366 (1946 , 2887)** | **1.9 (1.6 , 2.3)** | **700.3 (550.5 , 882.1)** | **3859 (2757 , 5297)** | **1.4 (1 , 1.8)** | **227.4 (159.7 , 320.2)** | **-67.5 (-77.6 , -54.7)** | **-3.88 (-4.21 , -3.55)** |
| **Egypt** | **110698 (73053 , 160960)** | **0.4 (0.3 , 0.6)** | **309.9 (200.7 , 455.5)** | **221195 (135795 , 354970)** | **0.8 (0.5 , 1.3)** | **290.3 (177 , 457.1)** | **-6.3 (-45.9 , 58)** | **-0.26 (-0.68 , 0.15)** |
| **Iran (Islamic Republic of)** | **50140 (38540 , 64703)** | **0.2 (0.2 , 0.3)** | **125.1 (96.7 , 159.7)** | **139833 (109766 , 175225)** | **0.7 (0.6 , 0.9)** | **158.9 (124.4 , 200.3)** | **27 (3.6 , 59.5)** | **0.82 (0.56 , 1.09)** |
| **Iraq** | **42143 (28971 , 57078)** | **0.5 (0.4 , 0.7)** | **372 (246.7 , 519.6)** | **54549 (35290 , 82758)** | **0.5 (0.4 , 0.8)** | **143 (88 , 225.5)** | **-61.5 (-76.7 , -37.9)** | **-3.25 (-3.45 , -3.05)** |
| **Jordan** | **3454 (2344 , 5004)** | **0.3 (0.2 , 0.5)** | **138.9 (89.4 , 211.6)** | **12615 (8213 , 17650)** | **0.6 (0.4 , 0.8)** | **121.4 (77.1 , 176.4)** | **-12.6 (-43 , 36.8)** | **-0.44 (-0.66 , -0.23)** |
| **Kuwait** | **1170 (858 , 1543)** | **0.3 (0.2 , 0.4)** | **76.3 (60.1 , 95.4)** | **3363 (2394 , 4598)** | **0.4 (0.3 , 0.6)** | **68.1 (49.9 , 90)** | **-10.8 (-24.5 , 4)** | **-0.41 (-0.90 , 0.09)** |
| **Lebanon** | **14570 (10974 , 18716)** | **1.2 (0.9 , 1.5)** | **535.2 (403.4 , 689.5)** | **10835 (7429 , 15522)** | **0.8 (0.6 , 1.1)** | **203.8 (138.8 , 291.2)** | **-61.9 (-73.9 , -47.1)** | **-3.13 (-3.31 , -2.94)** |
| **Libya** | **3292 (2514 , 4217)** | **0.2 (0.2 , 0.3)** | **120.3 (91.9 , 153.4)** | **11185 (7345 , 15020)** | **0.7 (0.5 , 0.8)** | **158.8 (103.5 , 214.5)** | **32.1 (-8.2 , 87.4)** | **0.97 (0.41 , 1.54)** |
| **Morocco** | **62478 (47492 , 79274)** | **0.6 (0.4 , 0.7)** | **324.8 (244.3 , 418.8)** | **58610 (40044 , 82346)** | **0.6 (0.4 , 0.8)** | **157.6 (107.7 , 222.5)** | **-51.5 (-65.2 , -32.1)** | **-2.49 (-2.69 , -2.29)** |
| **Oman** | **2065 (1339 , 3018)** | **0.3 (0.2 , 0.5)** | **139.9 (85.2 , 209.7)** | **6057 (4026 , 8578)** | **0.7 (0.5 , 1)** | **125.8 (79.5 , 182.1)** | **-10.1 (-34.1 , 29.2)** | **-0.35 (-0.63 , -0.07)** |
| **Palestine** | **3238 (2053 , 4740)** | **0.4 (0.3 , 0.6)** | **281.5 (171.4 , 423.4)** | **9873 (7511 , 12534)** | **1 (0.8 , 1.2)** | **288.9 (215.2 , 375.2)** | **2.6 (-30.4 , 61.3)** | **0.09 (-0.22 , 0.40)** |
| **Qatar** | **1144 (865 , 1547)** | **1.1 (0.9 , 1.5)** | **348.8 (245.6 , 484.4)** | **5715 (4097 , 7660)** | **1.3 (1 , 1.6)** | **218.1 (152.5 , 296.2)** | **-37.5 (-58.5 , -8.7)** | **-1.66 (-1.96 , -1.36)** |
| **Saudi Arabia** | **29380 (13272 , 48838)** | **0.5 (0.3 , 0.9)** | **307.4 (128.6 , 548.2)** | **50542 (30270 , 81200)** | **0.6 (0.4 , 0.9)** | **141.3 (86.8 , 233.6)** | **-54 (-70.6 , -20.9)** | **-2.65 (-2.83 , -2.46)** |
| **Sudan** | **47153 (24975 , 71454)** | **0.3 (0.1 , 0.4)** | **372.4 (187.7 , 575.3)** | **29763 (21608 , 39519)** | **0.2 (0.2 , 0.3)** | **105 (75.4 , 144.5)** | **-71.8 (-83.3 , -48.3)** | **-4.35 (-4.81 , -3.88)** |
| **Syrian Arab Republic** | **23482 (17135 , 31725)** | **0.5 (0.4 , 0.7)** | **316.4 (223.7 , 434.8)** | **24012 (15482 , 35496)** | **0.6 (0.4 , 0.8)** | **172.9 (112.1 , 256.6)** | **-45.4 (-62.6 , -24)** | **-2.11 (-2.39 , -1.82)** |
| **Tunisia** | **15961 (12157 , 20132)** | **0.6 (0.5 , 0.7)** | **219.7 (164.1 , 281.1)** | **34614 (24314 , 47583)** | **1.2 (0.9 , 1.5)** | **271.1 (189.9 , 371.3)** | **23.4 (-10.1 , 70.9)** | **0.75 (0.58 , 0.92)** |
| **Turkey** | **154232 (116685 , 198959)** | **0.7 (0.5 , 0.8)** | **315.5 (230.5 , 410.8)** | **194539 (143449 , 256399)** | **1 (0.8 , 1.2)** | **208.7 (153.1 , 276.7)** | **-33.8 (-50.8 , -11.1)** | **-1.50 (-1.79 , -1.20)** |
| **United Arab Emirates** | **9282 (7096 , 11935)** | **2.1 (1.7 , 2.7)** | **776.2 (561.1 , 1051.8)** | **34097 (22366 , 49773)** | **1.6 (1.1 , 2.2)** | **342 (204.3 , 523.2)** | **-55.9 (-73.3 , -31.8)** | **-2.81 (-3.17 , -2.44)** |
| **Yemen** | **39019 (26749 , 54030)** | **0.3 (0.2 , 0.5)** | **555.1 (378.4 , 775.9)** | **45794 (30451 , 64918)** | **0.4 (0.3 , 0.5)** | **211.5 (137.3 , 303.9)** | **-61.9 (-74.6 , -44.1)** | **-3.26 (-3.41 , -3.11)** |
